# Melatonin alleviates airway inflammation and anxiety-depression in asthma via gut microbiota–SCFA axis-mediated inhibition of microglial activation

**DOI:** 10.3389/fimmu.2026.1763305

**Published:** 2026-03-11

**Authors:** Jiahao Lai, Yue Wang, Liwan Zeng, Qinglin Deng, Youping Qiao, Jinyu Liao, Chaoqun Sun, Yan Geng, Huazhuo Wu, Dan Huang, Xuanna Zhao, Dong Wu

**Affiliations:** Department of Respiratory and Critical Care Medicine, Affiliated Hospital of Guangdong Medical University, Zhanjiang, China

**Keywords:** anxiety-depression comorbidity, asthma, gut microbiota, melatonin, short-chain fatty acids

## Abstract

**Background:**

Asthma frequently co-occurs with anxiety and depression, yet the mechanisms underlying this lung-brain comorbidity remain elusive. The gut-lung-brain axis has emerged as a potential key mediator.

**Methods:**

Using an ovalbumin (OVA)-induced murine asthma model, we administered melatonin or sodium butyrate via drinking water. We assessed airway inflammation, lung function, anxiety- and depression-like behaviors, gut microbiota composition, short-chain fatty acid (SCFA) levels, and the MAPK/P65/NLRP3 signaling pathway in the hippocampus and BV2 microglial cells. Fecal microbiota transplantation (FMT) and antibiotic depletion experiments were conducted to establish causality.

**Results:**

Both melatonin and sodium butyrate significantly alleviated airway inflammation, improved lung function, and ameliorated anxiety- and depression-like behaviors in asthmatic mice. Melatonin increased gut-derived butyrate levels and restored gut microbiota balance. FMT from melatonin-treated donors replicated the therapeutic benefits, whereas antibiotic-mediated microbiota depletion abrogated the effects of melatonin. Mechanistically, both treatments inhibited the activation of the MAPK/P65/NLRP3 pathway in hippocampal microglia and LPS-stimulated BV2 cells.

**Conclusion:**

Our findings demonstrate that melatonin mitigates asthma-related airway inflammation and neuropsychiatric comorbidity by modulating the gut microbiota-SCFA axis and suppressing microglial activation via the MAPK/P65/NLRP3 pathway. This study highlights a novel systemic mechanism and potential therapeutic strategy for asthma and its comorbidities.

## Introduction

Asthma is a prevalent chronic inflammatory airway disorder, characterized by airway hyperresponsiveness, reversible airflow obstruction, and excessive mucus production (1, 2). On a global scale, the prevalence and burden of asthma remain significantly high, presenting substantial challenges to public health systems (3). Increasing evidence indicates a strong association between asthma and psychiatric disorders, particularly anxiety and depression (4, 5). Clinical studies have reported that more than one-third of individuals with asthma exhibit symptoms of anxiety or depression. For example, a study involving 614 patients with persistent asthma (comprising 27.2% adolescents and 72.8% adults) found that approximately 36% of participants were diagnosed with anxiety or depressive disorders (6). Additionally, previous research has identified alterations in gut microbial composition and reduced levels of short-chain fatty acids (SCFAs) in both asthma patients and murine models of asthma (7). Similarly, gut microbiota dysbiosis has been implicated in the pathogenesis of neuropsychiatric conditions, including anxiety and depression (8). Collectively, these findings indicate that impaired production of microbiota-derived SCFAs may constitute a crucial mechanistic link between airway inflammation in asthma and the manifestation of anxiety and depression.

The gut microbiota, recognized as the largest micro-ecosystem within the human body, plays a pivotal role in modulating systemic immunity and central nervous system function through its metabolites (9). Among these metabolites, SCFAs—such as acetate, propionate, and butyrate—are generated via microbial fermentation of dietary fibers. These SCFAs are vital not only for maintaining intestinal barrier integrity and immune homeostasis but also for entering systemic circulation, where they exert significant anti-inflammatory effects throughout the body (10). Notably, research has demonstrated that both asthma patients and animal models exhibit gut microbiota dysbiosis, which is accompanied by markedly reduced levels of SCFAs (11, 12). Concurrently, a deficiency in SCFAs has been shown to contribute to the development of anxiety- and depression-like behaviors (13). Therefore, we propose the hypothesis that the disruption of the gut microbiota–SCFA axis may serve as a pivotal mechanism linking airway inflammation in asthma with its neuropsychiatric comorbidities.

Melatonin, a molecule synthesized by the pineal gland, is recognized for its dual role in regulating circadian rhythms and exerting potent immunomodulatory effects. It has been shown to effectively alleviate allergic airway inflammation while also demonstrating anxiolytic and antidepressant properties (14–17). Notably, accumulating evidence suggests that melatonin can substantially alter the gut microbial community structure and promote the growth of beneficial bacteria such as Lactobacillus (18, 19). This raises an important question: are the coordinated therapeutic effects of melatonin on asthma and its emotional comorbidities mediated through the regulation of the gut microbiota and the subsequent enhancement of endogenous SCFA biosynthesis? This hypothesis offers a novel perspective for understanding the systemic benefits of melatonin, although direct experimental evidence remains limited.

Based on this background, this study aimed to systematically investigate whether melatonin exerts synergistic anti-inflammatory and mood-enhancing effects by modulating the “gut-lung axis” and “gut-brain axis” via the “gut microbiota-SCFA” axis. We further sought to elucidate the role of the central MAPK/NLRP3 signaling pathway in this process.

## Materials and methods

### Experimental animals

Six-week-old female C57BL/6 mice were purchased from Guangdong Yaokang Bio-technology Co., Ltd. All mice were housed under specific pathogen-free (SPF) conditions with a 12-hour light/dark cycle. All animal experiments were approved by the Institutional Animal Care and Use Committee (IACUC) of the Affiliated Hospital of Guangdong Medical University (Approval No.: AHGDMU-LAC-B-202510-0095) and were conducted in strict accordance with the National Guidelines for the Care and Use of Laboratory Animals. All procedures were performed in compliance with the ARRIVE guidelines. Mice were randomly assigned to experimental groups.

### Asthma modeling and therapeutic intervention

According to the previous studies (20), an ovalbumin (OVA)-induced asthma model was established in mice through sensitization and challenge. Sensitization was performed by intraperitoneal injection of 200 μl PBS suspension containing 50 μg OVA and 2 mg aluminum hydroxide adjuvant on days 0, 7, and 14. From day 15 onward, airway challenge was conducted daily for 7 consecutive days using 5% OVA solution via nebulization for 30 minutes per session. During the challenge period, therapeutic interventions were administered. To minimize handling stress associated with repeated injections, which can confound behavioral and inflammatory outcomes, melatonin (100 mg/kg/day) (Sigma, M5250-5G)and sodium butyrate (1 mg/kg/day) were administered via drinking water. The melatonin solution was prepared in light-protected bottles at a final concentration of 0.3 mg/mL, while sodium butyrate was administered at 3 μg/mL. Both drug solutions were freshly prepared and replaced daily to ensure stability and accurate dosing. (**Supplementary Tables 1**, **2**).

### Antibiotic combination treatment

Following previously described methods (21), C57BL/6 mice were administered an antibiotic mixture (ABX) dissolved in drinking water *ad libitum* for 7 consecutive days. The antibiotic cocktail consisted of ampicillin (1 g/L), neomycin sulfate (1 g/L), metronidazole (1 g/L), and vancomycin (0.5 g/L). The antibiotic-containing drinking solution was replaced every two days with freshly prepared solution. After the 7-day treatment period, the antibiotic water was replaced with regular drinking water.

### Fecal sample collection and fecal microbiota transplantation

For FMT administration, fresh stools were collected from mice based on previous reports (21–23). Fecal samples were immediately collected into sterile EP tubes placed on ice and stored at -80 °C until further use. For FMT administration, fresh fecal samples were collected from donor mice according to previously established protocols. Female C57BL/6 mice were maintained under identical housing and environmental conditions. All donor fecal pellets were collected under specific pathogen-free (SPF) conditions. Fresh feces were immediately suspended in sterile saline (100 mg/mL) and incubated for 15 minutes. The dissolved fecal samples were then vortexed and centrifuged at 1000 × g for 5 minutes at 4 °C. The supernatant was collected under sterile conditions and stored at -80 °C until FMT administration. For transplantation, each recipient mouse received 100 μL of the bacterial suspension or vehicle (saline) daily via oral gavage for a duration of 2 weeks.

### Behavioral analysis

All behavioral tests were conducted within 24 hours following the final OVA challenge. Testing was performed in a sound-attenuated behavioral laboratory with uniform illumination. All apparatus were thoroughly cleaned with 75% ethanol after each trial to eliminate olfactory cues. To minimize potential carry-over effects, the behavioral tests were performed in the following order from least to most stressful: Open Field Test (OFT), Forced Swim Test (FST), and Tail Suspension Test (TST). The order of mice being tested within each assay was randomized across experimental groups.

### Tail suspension test

The tail suspension test was employed to evaluate depressive−like behavior. Mice were suspended approximately 2 cm from the tip of the tail using adhesive tape attached to a bracket inside a tail suspension chamber (50 × 50 × 50 cm), with the head positioned about 15 cm from the bottom. The test lasted 6 min; during the final 3 min, immobility time, struggling time (defined as vigorous, escape−directed movements of the limbs and body), shaking time (small, non−directed movements) were recorded and analyzed. Immobility was defined as the absence of any active limb or body motion, with the mouse remaining completely passive. A significant increase in immobility time, accompanied by reductions in struggling and/or shaking time, was interpreted as a depressive−like behavioral phenotype.

### Forced swim test

The forced swim test was used to further assess depressive−like behavior. Each mouse was placed individually in a transparent cylindrical container (25 cm height × 15 cm diameter) filled with water maintained at 23–25 °C. The water depth was sufficient to prevent the mouse from touching the bottom with its hind limbs. The test duration was 6 min; during the final 3 min, immobility time, struggling time (defined as vigorous, escape−directed limb and body movements), and shaking time (active, non−struggling movements to maintain head above water) were recorded. Immobility was defined as the cessation of active struggling or swimming, with the mouse displaying only minimal movements necessary to keep its head above water. A significant increase in immobility time, along with decreases in struggling and/or shaking time, was interpreted as indicative of a depressive−like behavioral phenotype.

### Open field test

The open field test was used to assess locomotor activity and anxiety-like behavior. Each mouse was placed individually in the center of a square arena (40 × 40 × 40 cm) and allowed to explore freely for 10 minutes. Movement trajectories were recorded and analyzed using a video tracking system. The arena was divided into a center zone (defined as the area >10 cm from the walls) and a peripheral zone. The following parameters were quantified: total distance traveled, distance moved in the center zone, movement time, and rest time. Decreased total distance traveled and increased rest time were interpreted as indicators of anxiety-like behavior.

### Respiratory mechanics

Invasive pulmonary function testing in mice is typically performed under deep anesthesia. The mouse is first placed in a supine position and secured, followed by shaving and disinfecting the neck area. A midline incision is made along the skin of the neck, and subcutaneous tissue is bluntly dissected to expose the trachea. A small incision is then made between the tracheal cartilage rings, through which a tracheal cannula is quickly inserted and secured. The cannula is immediately connected to the ventilator port of a pulmonary function instrument such as the flexiVent. Once breathing stabilizes, standardized ventilation, pressure-volume loop measurements, and forced oscillation technique-based frequency scans are sequentially conducted via software control to obtain key parameters including airway resistance, lung compliance, and lung elasticity. Throughout the procedure, anesthesia depth and vital signs must be strictly maintained and monitored.

### Microglial cells isolation and extraction

Microglial cells were isolated using a combination of enzymatic-mechanical digestion and Percoll density gradient centrifugation. Briefly, anesthetized mice were perfused transcardially with ice-cold PBS to remove blood. Whole brains were rapidly dissected, and the meninges were carefully removed in chilled D-Hanks’ Balanced Salt Solution. The tissue was then mechanically minced into approximately 1 mm³ fragments. Tissue fragments were digested with 0.25% trypsin at 37 °C for 15–20 minutes, and the digestion was terminated by adding DMEM/F12 complete medium supplemented with 10% fetal bovine serum. The cell suspension was filtered through a 70 μm cell strainer to obtain a single-cell suspension. Subsequently, the single-cell suspension was gently layered onto an equal volume of Percoll separation solution (density: 1.030–1.070 g/mL) and centrifuged at 800 × g for 20 minutes at 4 °C without brake. After centrifugation, the cell layer located between the intermediate myelin layer and the bottom erythrocyte layer was carefully collected, representing the enriched microglial fraction.

### Gut microbiome analysis

Mouse colonic content samples were stored at -80 °C until use. Total microbial DNA was extracted using the HiPure Stool DNA Mini Kit (Magen), and its concentration and quality were assessed by Qubit 4.0 and agarose gel electrophoresis. The V3–V4 region of the bacterial 16S rRNA gene was amplified with primers 338F/806R using 2× TransStart FastPfu PCR SuperMix. Purified amplicons were subjected to PE250 sequencing on the Illumina NovaSeq 6000 platform. Raw data were processed in QIIME2 (v2022.8) for quality control, denoising, and amplicon sequence variant (ASV) clustering. Taxonomic assignment was performed using the RDP Classifier against the SILVA 138.1 database with a confidence threshold of 0.7. All statistical analyses and visualizations were conducted in the R environment using packages such as vegan and phyloseq for alpha diversity, beta diversity (Bray–Curtis distance, PERMANOVA test), and intergroup.

### SCFA analysis by GC–MS

Colonic content samples were homogenized in 1.5 mL tubes with 500 μL of ultrapure water and 100 mg of glass beads, followed by centrifugation at 4 °C and 12,000 rpm for 10 min. A 200 μL aliquot of the supernatant was mixed with 100 μL of 15% phosphoric acid, 20 μL of internal standard (4-methylvaleric acid, 375 μg/mL), and 280 μL of ethyl ether. After vortexing and recentrifugation, the supernatant was analyzed by GC–MS. Analysis was performed on a Thermo TRACE 1300 gas chromatography system coupled with an ISQ 7000 mass spectrometer, equipped with an Agilent HP-INNOWAX capillary column (30 m × 0.25 mm, 0.25 μm). Injection volume was 1 μL with a split ratio of 10:1, using helium as carrier gas at 1.0 mL/min. The temperature program was as follows: initial 90 °C, increased to 120 °C at 10 °C/min, then to 150 °C at 5 °C/min, and finally to 250 °C at 25 °C/min held for 2 min. Mass detection utilized electron impact ionization (70 eV) in selected ion monitoring (SIM) mode. Quantification of SCFAs (acetate, propionate, butyrate, isobutyrate, valerate, isovalerate, caproate) was based on internal standard calibration using a series of standard concentrations.

### Microglial cells transcriptome sequencing and analysis

Total RNA from microglial cells was extracted using an Animal Total RNA Extraction Kit (DP431), with quality verified by agarose gel electrophoresis and Qubit quantification. mRNA was enriched with Oligo d(T) beads, and strand-specific libraries were constructed using the U-mRNAseq Library Prep Kit. Sequencing was performed on the Illumina NovaSeq 6000 platform (PE150). Raw reads were quality-controlled with fastp, aligned to the mouse reference genome (GRCm38) using HISAT2, and assembled and quantified with StringTie. Gene expression levels were reported as FPKM. Differential expression analysis was conducted using edgeR with thresholds of |log2FC| ≥ 1 and adjusted p-value (padj) < 0.05. Functional enrichment analysis of differentially expressed genes was performed for GO, KEGG, and Reactome databases (p < 0.05), and co-expression networks were constructed using WGCNA.

### Histological examination

Tissue sections were prepared by Wuhan Servicebio Technology Co., Ltd. Lung tissues were fixed in 4% paraformaldehyde, dehydrated, embedded in paraffin, and sectioned at 4 μm thickness. The sections were stained with: 1) hematoxylin and eosin (H&E) for morphological assessment; 2) Masson’s trichrome for collagen deposition; and 3) periodic acid–Schiff (PAS) for carbohydrate detection. Images were captured using an Olympus microscope.

### Immunofluorescence staining and image analysis

Brain tissues were fixed in 4% paraformaldehyde, dehydrated through graded ethanol, embedded in paraffin, and sectioned at 5 μm thickness. The sections were subjected to Nissl staining with 0.5% cresyl violet to assess neuronal morphology and survival in the hippocampal CA1and medial prefrontal cortex (mPFC). For immunofluorescence staining, sections were deparaffinized, rehydrated, and subjected to antigen retrieval, followed by blocking with 5% bovine serum albumin. Sections were then incubated with primary antibodies against TMEM119 (microglia) overnight at 4 °C, followed by incubation with fluorescence-conjugated secondary antibodies. Nuclei were counterstained with DAPI. All images were captured using a fluorescence microscope (Olympus) and analyzed with ImageJ software.

### Cell culture and treatment

The murine microglial cell line BV-2 was used in this study. The BV-2 cell line is a well-established and widely accepted *in vitro* model for studying microglial activation and neuroinflammatory pathways. As the primary objective of our *in vitro* experiments was to mechanistically validate the direct anti-inflammatory effects of melatonin and sodium butyrate on the MAPK/P65/NLRP3 pathway within microglia, the use of this standard model was deemed appropriate.

BV-2 cells (Catalog No. CL-0493), authenticated by short tandem repeat (STR) profiling, were purchased from Procell Life Science & Technology Co., Ltd. (Wuhan, China). Cells were maintained in MEM medium supplemented with 10% fetal bovine serum (FBS) and 1% penicillin-streptomycin at 37 °C in a humidified atmosphere of 5% CO_2_. For experiments, cells in the logarithmic growth phase were harvested and seeded at an appropriate density into culture plates. To avoid potential solvent interference in cell experiments, melatonin used for cell−based assays was sourced from OriLeaf (Catalog No. T94364). This product is directly soluble in DMSO and can be diluted to working concentrations with cell culture medium. To examine the protective effects of melatonin and sodium butyrate, cells were pretreated for 2 h with the indicated concentrations of melatonin (2.5, 5, and 10 μM) or sodium butyrate (2.5, 5, and 10 mM) before inflammatory stimulation. To elucidate the involvement of the MAPK signaling pathway, the p38 MAPK inhibitor SB203580 (10 μM) was applied 2 hours before lipopolysaccharide (LPS) exposure. Following pretreatment, inflammation was induced by stimulating the cells with 100 ng/mL LPS for 24 hours. After the treatment period, cell culture supernatants and cellular pellets were collected separately for subsequent analysis.

### Reagents and assay materials

Chemicals and reagents used in this study are listed in **Table 1**. Antibodies employed for Western blotting, immunohistochemistry, and immunofluorescence are detailed in **Table 2**. Primer sequences for quantitative PCR are provided in **Table 3**.

**Table 1:** Chemicals and reagents.

Chemicals and reagents	Brand	Catalog no
Ovalbumin (OVA)	Sangon Biotech Co., Ltd.	A605084–0025
Aluminum hydroxide	Sigma	239,186
Melatonin	Sigma	M5250-5G
Melatonin	OriLeaf	T94364
LPS	Sigma	L2880
SB203580	Selleck Chemicals	S1076
IL-1β ELISA kits	NeoBioscience Technology Co., Ltd.	EMC001b.96
IL-6 ELISA kits	NeoBioscience Technology Co., Ltd.	EMC004QT.96

**Table 2:** Antibodies used for western blotting, immunohistochemistry, and immunofluorescence.

Antibodies	Brand	Catalog no	Applications
β-actin Mouse Polyclonal	Proteintech	2D24H5	WB: 1:2000
MAPK Family Antibody Sampler Kit	CST	9926T	WB: 1:2000 IF:1:200
Mouse Reactive Inflammasome Antibody Sampler Kit	CST	20836T	WB: 1:2000 IF:1:200
P65	CST	8242S	WB: 1:2000
p-P65	CST	3033S	WB: 1:1000
p-P65	Beyotime	AF5875	IF:1:200
TMEM119	Proteintech	27585-1-AP	IF:1:200

**Table 3:** Gene -specific primer sequences used in this study.

Genes	Forward (5′ to 3′)	Reverse (5′to 3′)
IL-6	TAGTCCTTCCTACCCCAATTTCC	TTGGTCCTTAGCCACTCCTTC
IL-1β	GCAACTGTTCCTGAACTCAACT	ATCTTTTGGGGTCCGTCAACT
β-actin	AGTGTGACGTTGACATCCGT	GCAGCTCAGTAACAGTCCGC

### Experimental design and reuse of control groups

**Figure 1:**
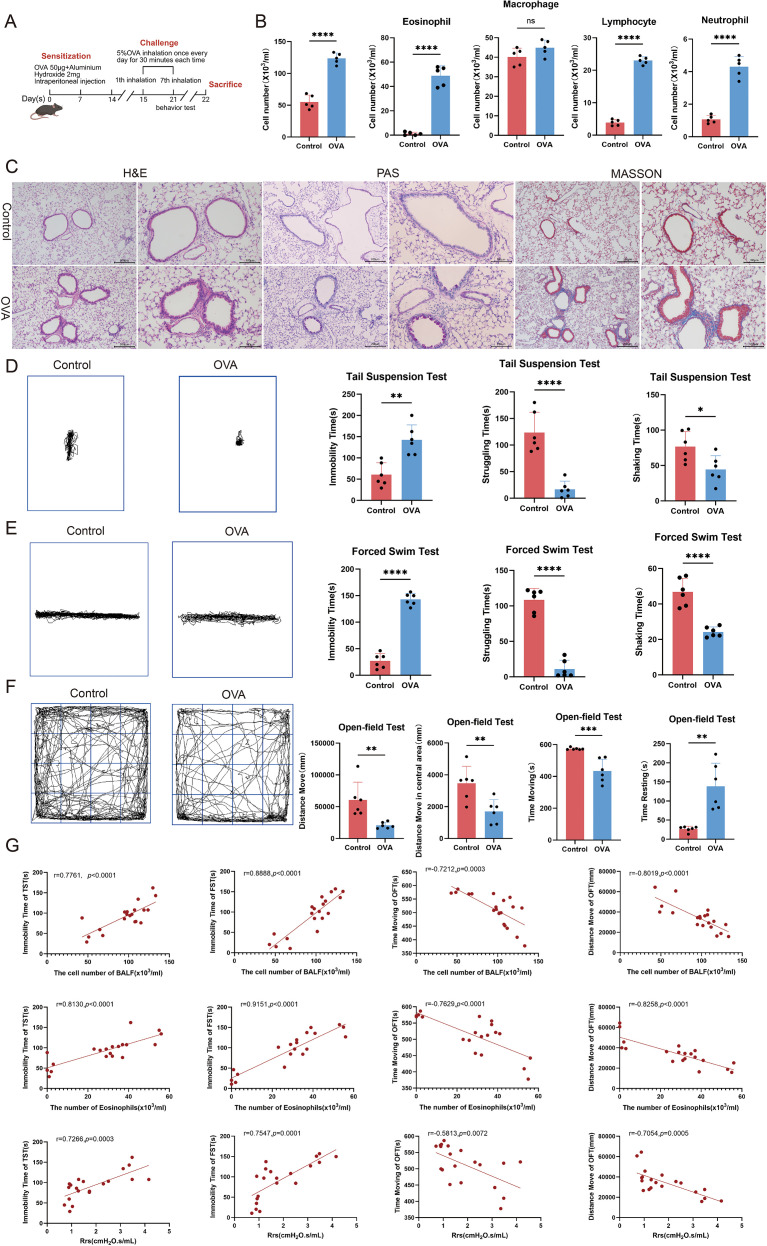
Airway inflammation positively correlates with anxiety- and depressive-like behaviors in asthmatic mice. **(A)** Schematic of the OVA-induced allergic asthma model. **(B)** Numbers of total cells and inflammatory cells in BALF (n=5). **(C)** Representative H&E, PAS, and Masson’s trichrome staining of lung tissues (left scale bar: 200 μm; right scale bar: 100 μm). **(D, E)** Representative movement trajectories and quantification of immobility, struggling, and shaking time in TST and FST (n=6). **(F)** Representative movement trajectories and quantification of total distance, center distance, movement time, and rest time in OFT (n=6). **(G)** Correlations between airway inflammation (total cells and eosinophils in BALF, and pulmonary airway resistance) and behavioral parameters (immobility time in TST/FST and total distance moved/movement time in OFT) (n = 20). Data are presented as mean ± SEM. ns, not significant (*p* > 0.05), *p < 0.05, **p < 0.01, ***p < 0.001, ****p < 0.0001.

**Figure 2:**
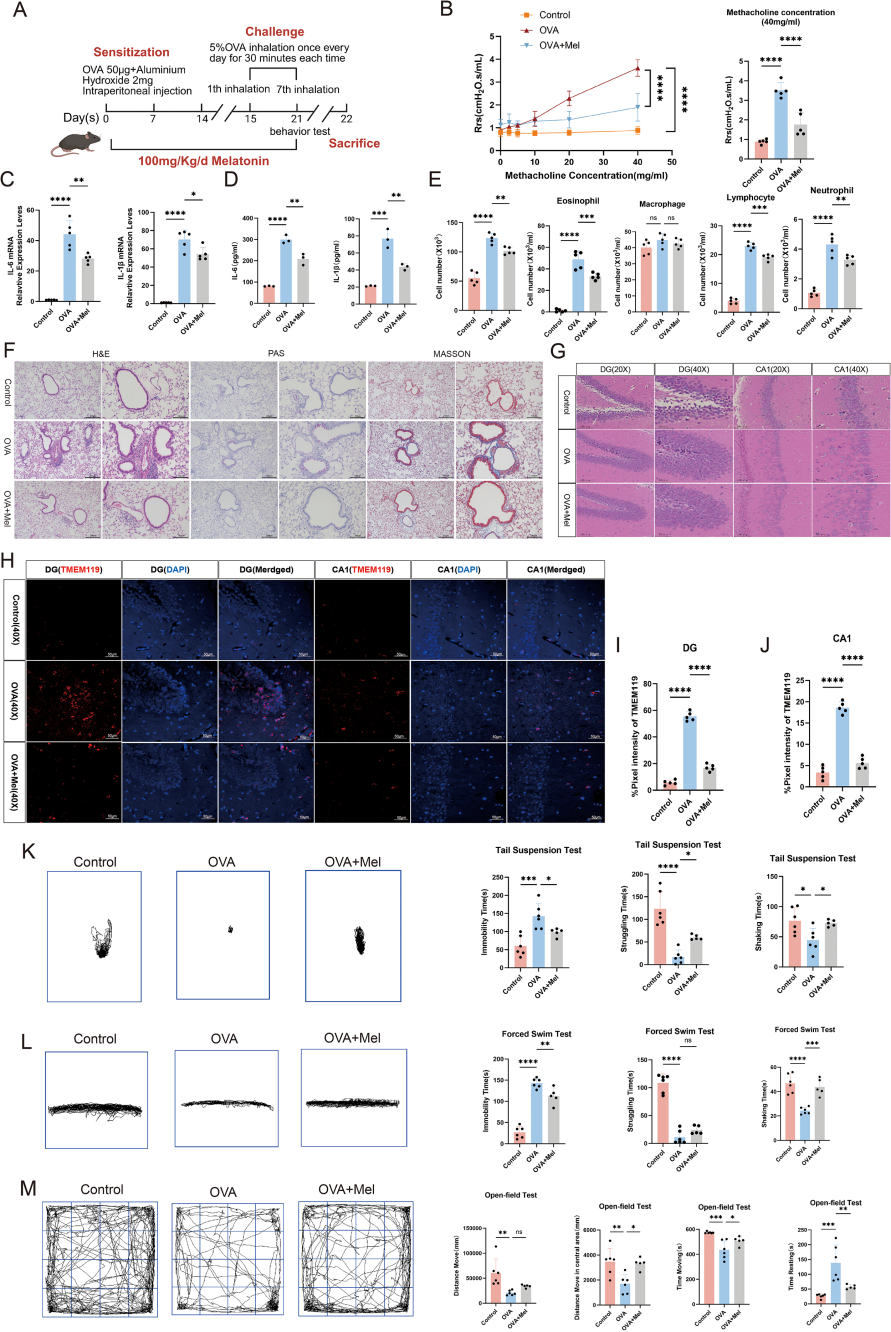
Melatonin ameliorates airway inflammation and anxiety-depressive behaviors in asthmatic mice. **(A)** Schematic of melatonin treatment in allergic asthma. **(B)** Airway responsiveness assessed by methacholine challenge test (40 mg/mL) (n=5). **(C)** mRNA levels of IL-6 and IL-1β in lung tissue measured by qPCR (n=5). **(D)** Protein levels of IL-1β and IL-6 in BALF measured by ELISA (n=3). **(E)** Numbers of total cells and inflammatory cells in BALF (n=5). **(F)** Representative H&E, PAS, and Masson’s trichrome staining of lung tissues (left scale bar: 200 μm; right scale bar: 100 μm). **(G)** Representative H&E staining of the hippocampal dentate gyrus (DG) and CA1 region (20X scale bar:100μm; 40X scale bar: 50 μm). **(H-J)** Representative immunofluorescence staining and quantitative analysis of TMEM119 in the DG and CA1 regions (scale bar: 50 μm). **(K, L)** Representative movement trajectories and quantification of immobility, struggling, and swinging time in TST and FST (Control and OVA: n=6; OVA+Mel: n=5). **(M)** Representative movement trajectories and quantification of total distance, center distance, movement time, and rest time in OFT (Control and OVA: n=6; OVA+Mel: n=5). Data are presented as mean ± SEM. ns, not significant (*p* > 0.05), *p < 0.05, **p < 0.01, ***p < 0.001, ****p < 0.0001.

**Figure 3:**
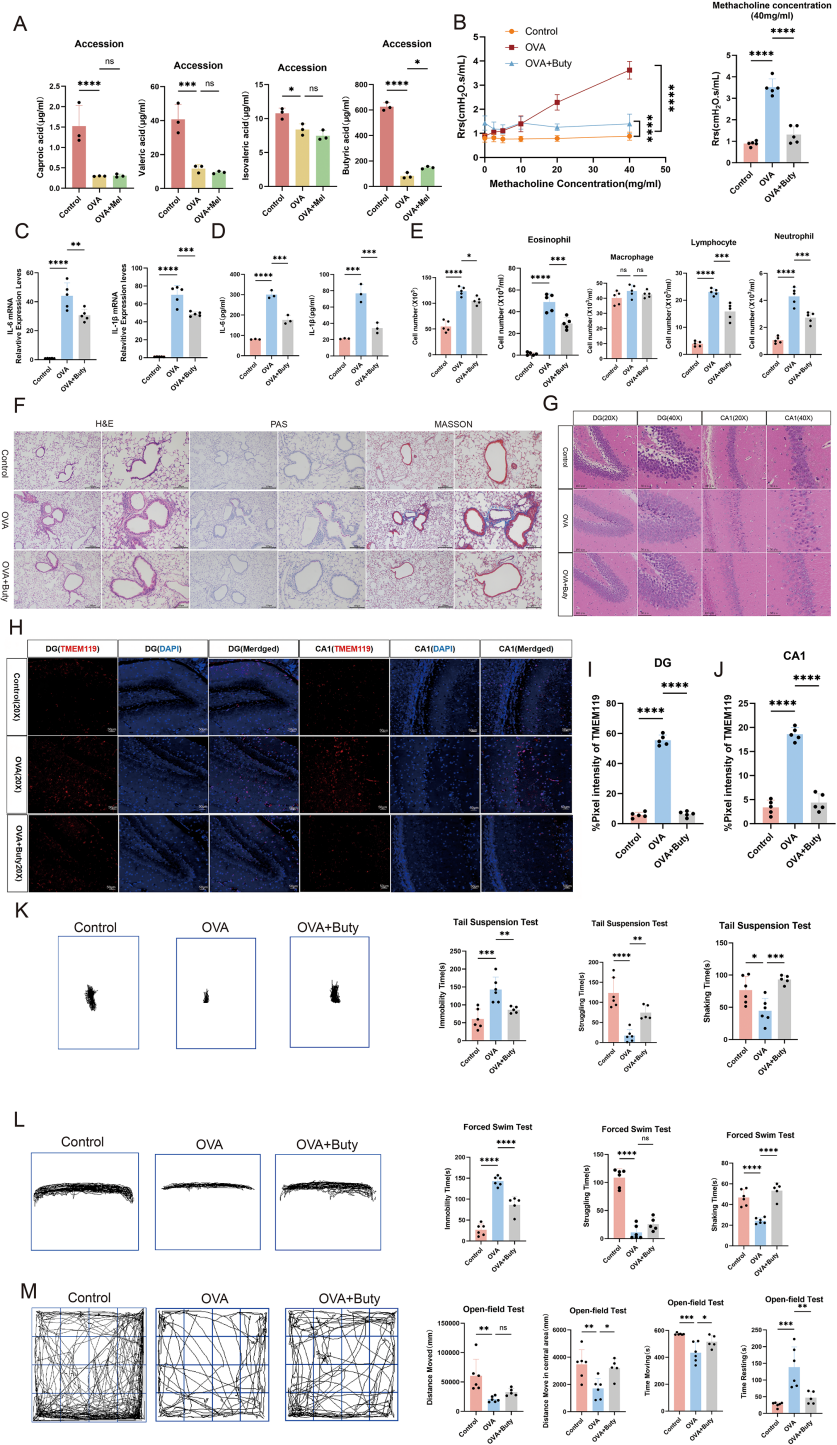
Sodium butyrate ameliorates airway inflammation and anxiety-depressive behaviors in asthmatic mice. **(A)** Targeted quantification of gut microbiota-derived SCFAs (n=3). **(B)** Airway responsiveness assessed by methacholine challenge test (40 mg/mL) (n=5). **(C)** mRNA levels of IL-6 and IL-1β in lung tissue measured by qPCR (n=5). **(D)** Protein levels of IL-1β and IL-6 in BALF measured by ELISA (n=3). **(E)** Total and differential inflammatory cell counts in BALF (n=5). **(F)** Representative H&E, PAS, and Masson’s trichrome staining of lung tissues (left scale bar: 200 μm; right scale bar: 100 μm). **(G)** Representative H&E staining of the hippocampal dentate gyrus (DG) and CA1 region (20X scale bar:100μm; 40X scale bar: 50 μm). **(H-J)** Representative immunofluorescence staining and quantitative analysis of TMEM119 in the DG and CA1 regions (scale bar: 50 μm). **(K, L)** Representative movement trajectories and quantification of immobility, struggling, and swinging time in TST and FST (Control and OVA: n=6; OVA+Buty: n=5). **(M)** Representative movement trajectories and quantification of total distance, center distance, movement time, and rest time in OFT (Control and OVA: n=6; OVA+Buty: n=5). Data are presented as mean ± SEM. ns, not significant (*p* > 0.05), *p < 0.05, **p < 0.01, ***p < 0.001, ****p < 0.0001.

To maximize the use of experimental animals and ensure consistency across comparative analyses, control and OVA-induced asthmatic groups from the same cohort of animals were shared across the initial phenotypic characterization experiments (**Figures 1**-**3**). This approach minimizes inter-batch variability when comparing multiple treatment conditions against the same baseline groups.

### Statistical analysis

All data were analyzed using Prism software (GraphPad, San Diego, CA, USA). Data are presented as mean ± standard error of the mean (SEM). Comparisons between two groups were performed using Student’s t-test. Multiple group comparisons were conducted by one-way analysis of variance (ANOVA) followed by Fisher’s least significant difference (LSD) test. A *p*-value < 0.05 was considered statistically significant.

## Results

### Reciprocal interaction between airway inflammation and anxiety-depressive behaviors in asthmatic mice

To investigate the interplay between asthma and anxiety-depression comorbidity, we successfully established a murine model of allergic asthma (**Figure 1A**). Compared with the control group, asthmatic mice exhibited significantly increased numbers of total cells and inflammatory cells in bronchoalveolar lavage fluid (BALF) (**Figure 1B**), except for macrophages, confirming successful induction of airway inflammation. Histopathological examination of lung tissues further revealed enhanced inflammatory cell infiltration, goblet cell hyperplasia, and pathological collagen deposition (**Figure 1C**).

At the behavioral level, asthmatic mice showed a significant increase in immobility time in both the TST and FST (**Figures 1D, E**), indicating depressive-like behaviors. Concurrently, these mice displayed marked anxiety-like behaviors in the OFT, characterized by reduced time spent in the center zone and decreased total travel distance (**Figure 1F**).

To elucidate the relationship between inflammation and behavioral alterations, correlation analysis was performed. Results demonstrated that total inflammatory cell and eosinophil counts in bronchoalveolar lavage fluid, as well as pulmonary airway resistance, showed positive correlations with depression-like behaviors (e.g., total inflammatory cell count vs. immobility time in FST: r = 0.8888, *p <*0.0001) and anxiety-like behaviors (e.g., total inflammatory cell count vs. total distance traveled in OFT: r = -0.8019, p <0.0001) (**Figure 1G**).

Collectively, these findings indicate that airway inflammation and anxiety-depressive behaviors not only coexist in asthmatic mice but also exhibit severity-dependent interrelationships, suggesting a link between pulmonary and neuropsychiatric pathologies.

### Melatonin alleviates airway inflammation and anxiety-depressive behaviors in asthmatic mice

In this study, we investigated the therapeutic effects of melatonin in a murine model of allergic asthma (**Figure 2A**). Compared with the asthma model group, melatonin treatment significantly improved lung function, as evidenced by reduced airway resistance (**Figure 2B**). Concurrently, melatonin markedly decreased total and differential inflammatory cell counts in BALF, except for macrophages. And also downregulated the expression of inflammatory cytokines IL-6 and IL-1β (**Figures 2C, D**). Furthermore, histological analysis demonstrated that melatonin attenuated inflammatory cell infiltration, suppressed goblet cell hyperplasia, and reduced pathological collagen deposition in lung tissues (**Figure 2F**). These findings collectively indicate that melatonin effectively alleviates airway inflammation in asthmatic mice.

We next examined whether the improvement in lung pathology was accompanied by behavioral and neurological benefits. Further histological examination of brain tissues revealed that melatonin treatment ameliorated pathological alterations in the hippocampal CA1 region and dentate gyrus, including attenuated thinning and disorganization of the cellular layer and reduced vacuolar degeneration (**Figure 2G**). Consistent with these improvements, decreased expression of the microglial marker TMEM119 indicated suppressed microglial activation (**Figures 2H–J**).

In behavioral tests, melatonin-treated mice showed significant amelioration of anxiety- and depressive-like behaviors. This was demonstrated by shortened immobility time and prolonged struggling/swinging time in both the TST and FST (**Figures 2K, L**), along with significantly increased center zone activity and total travel distance in the OFT (**Figure 2M**).

In summary, these results demonstrate that melatonin not only mitigates airway inflammation but also alleviates anxiety- and depressive-like behaviors in asthmatic mice, effects associated with improved hippocampal integrity and suppressed neuroinflammation.

### Sodium butyrate alleviates airway inflammation and anxiety-depressive behaviors in asthmatic mice

Consistent with previous findings (24), fecal metabolomic analysis revealed that melatonin intervention increased butyrate concentrations in asthmatic mice (**Figure 3A**), leading us to hypothesize that sodium butyrate (Buty) may be a key mediator of melatonin’s therapeutic effects. To test this, we administered Buty to asthmatic mice. Buty treatment significantly improved pulmonary function, as shown by reduced airway resistance (**Figure 3B**). It markedly decreased total and differential inflammatory cell counts in BALF, except for macrophages. And also downregulated the expression of inflammatory cytokines IL-6 and IL-1β (**Figures 3C–E**). Histological examination confirmed that Buty attenuated inflammatory cell infiltration, suppressed goblet cell hyperplasia, and reduced pathological collagen deposition in lung tissues (**Figure 3F**). These results collectively indicate that Buty effectively alleviates airway inflammation in asthmatic mice.

At the behavioral level, Buty treatment significantly ameliorated anxiety- and depressive-like behaviors. In the TST and FST, Buty-treated mice exhibited decreased immobility time and increased struggling/swinging time (**Figures 3K, L**). In the OFT, these mice displayed increased center zone activity, total travel distance, and movement time, along with decreased resting time (**Figure 3M**). These behavioral findings, which closely recapitulated the effects of melatonin, consistently demonstrate that Buty mitigates asthma-associated emotional comorbidities.

Further neurohistological evaluation revealed that Buty treatment also ameliorated pathological alterations in the hippocampal CA1 region and dentate gyrus, including attenuated thinning and disorganization of the cellular layer and reduced vacuolar degeneration (**Figure 3G**). Additionally, decreased expression of the microglial marker TMEM119 suggested suppression of microglial activation (**Figures 3H–J**). Collectively, these results establish that sodium butyrate alleviates anxiety- and depressive-like behaviors in asthmatic mice, supporting its role as a critical mediator in the gut-lung-brain axis.

### Effects of melatonin and sodium butyrate on gut microbiota diversity and structure in asthmatic mice

**Figure 4:**
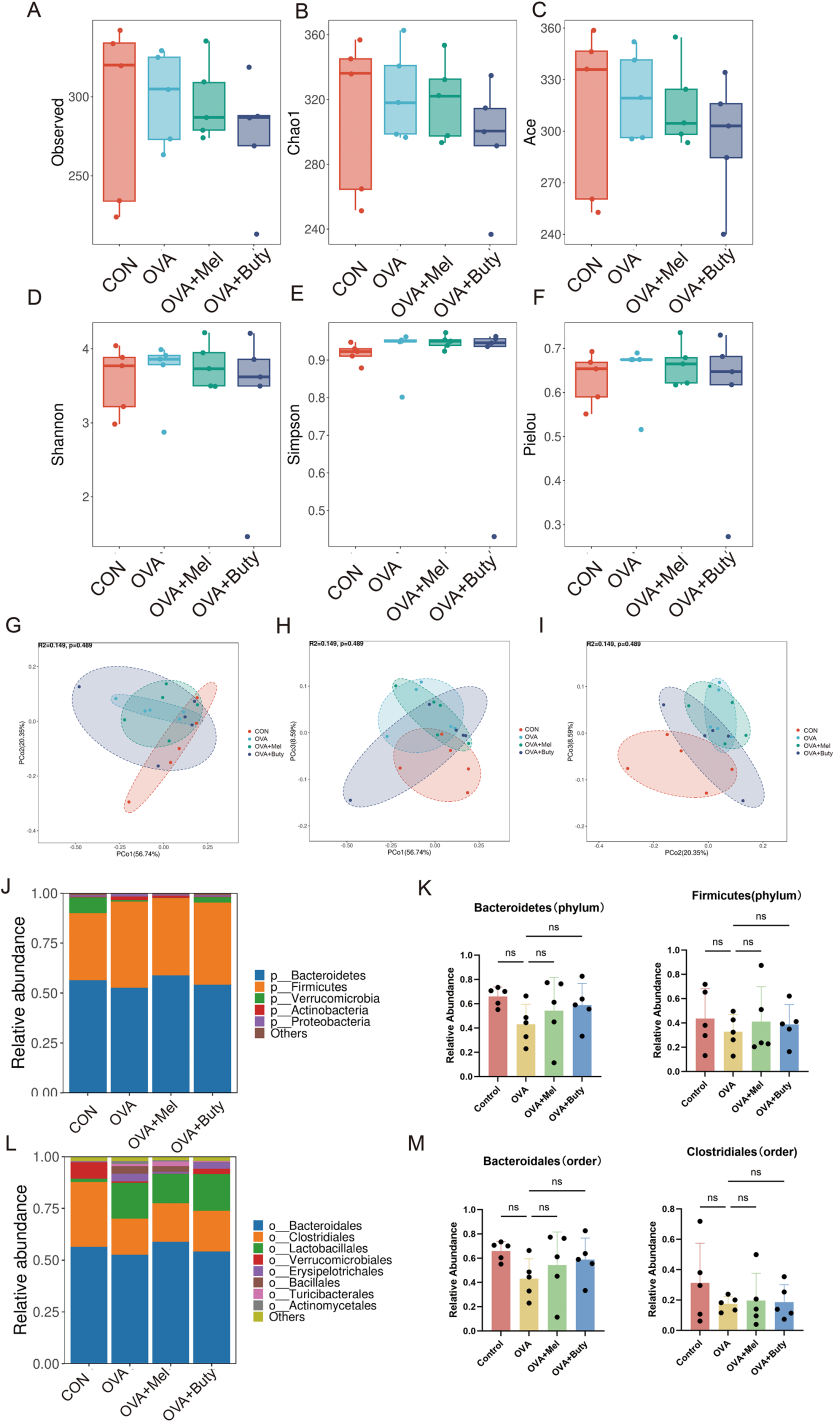
Effects of melatonin and sodium butyrate intervention on gut microbiota diversity and structural characteristics in asthmatic mice. **(A-F)** α-Diversity indices including Ace, Chao, Shannon, Simpson, and Pielou (n=5). **(G-I)** Principal coordinate analysis (PCoA) based on weighted UniFrac distance (n=5). **(J)** Relative abundance of the top 10 bacterial phyla (n=5). **(K)** Comparison of differentially abundant bacterial phyla among the top 10 phyla (n=5). **(L)** Relative abundance of the top 10 bacterial orders (n=5). **(M)** Comparison of differentially abundant bacterial orders among the top 10 orders (n=5). Data are presented as mean ± SEM. ns, not significant (*p* > 0.05).

To evaluate the impact of interventions on gut microbiota structure and composition, we performed 16S rRNA gene sequencing. α diversity analysis revealed that the OVA induced asthma group exhibited lower species richness (Ace and Chao indices) than the control group (**Figures 4B, C**). This decrease in richness was not restored by either melatonin or sodium butyrate treatment. Conversely, indices that incorporate both species richness and evenness (Shannon, Simpson, and Pielou) demonstrated that the OVA model also reduced microbial evenness. Treatment with melatonin or sodium butyrate resulted in improvement in these evenness related indices (**Figures 4D–F**), indicating that the interventions primarily facilitated structural rebalancing rather than merely increasing species numbers.

β diversity analysis (PCoA) showed separation among the OVA, control, and both treatment groups (**Figures 4G–I**). While the OVA group formed a distinct cluster away from the control group, both melatonin- and sodium butyrate-treated groups exhibited altered microbiota structures that were different from the OVA group, although the melatonin-treated group did not fully converge with the control cluster. This indicates that the interventions, modulated the dysbiotic microbiota but did not completely restore it to the healthy state.

At the phylum level, the relative abundances of Bacteroidetes and Firmicutes among the groups (**Figures 4J, K**), statistical analysis revealed no significant differences. Similarly, at the class level for Bacteroidia and Clostridia (**Figures 4L, M**), the relative abundances were comparable across all groups. No statistically significant differences were observed between the OVA group, the control group, and the treatment groups. This suggests that the apparent lack of significant differences at these taxonomic levels should be interpreted with caution. In summary, both melatonin and sodium butyrate interventions partially ameliorated the gut microbiota dysbiosis in asthmatic mice. However, the restorative effects on specific bacterial taxa were limited and will require confirmation in future studies with larger sample sizes.

### Fecal microbiota transplantation influences airway inflammation and anxiety-depressive behaviors in mice

**Figure 5:**
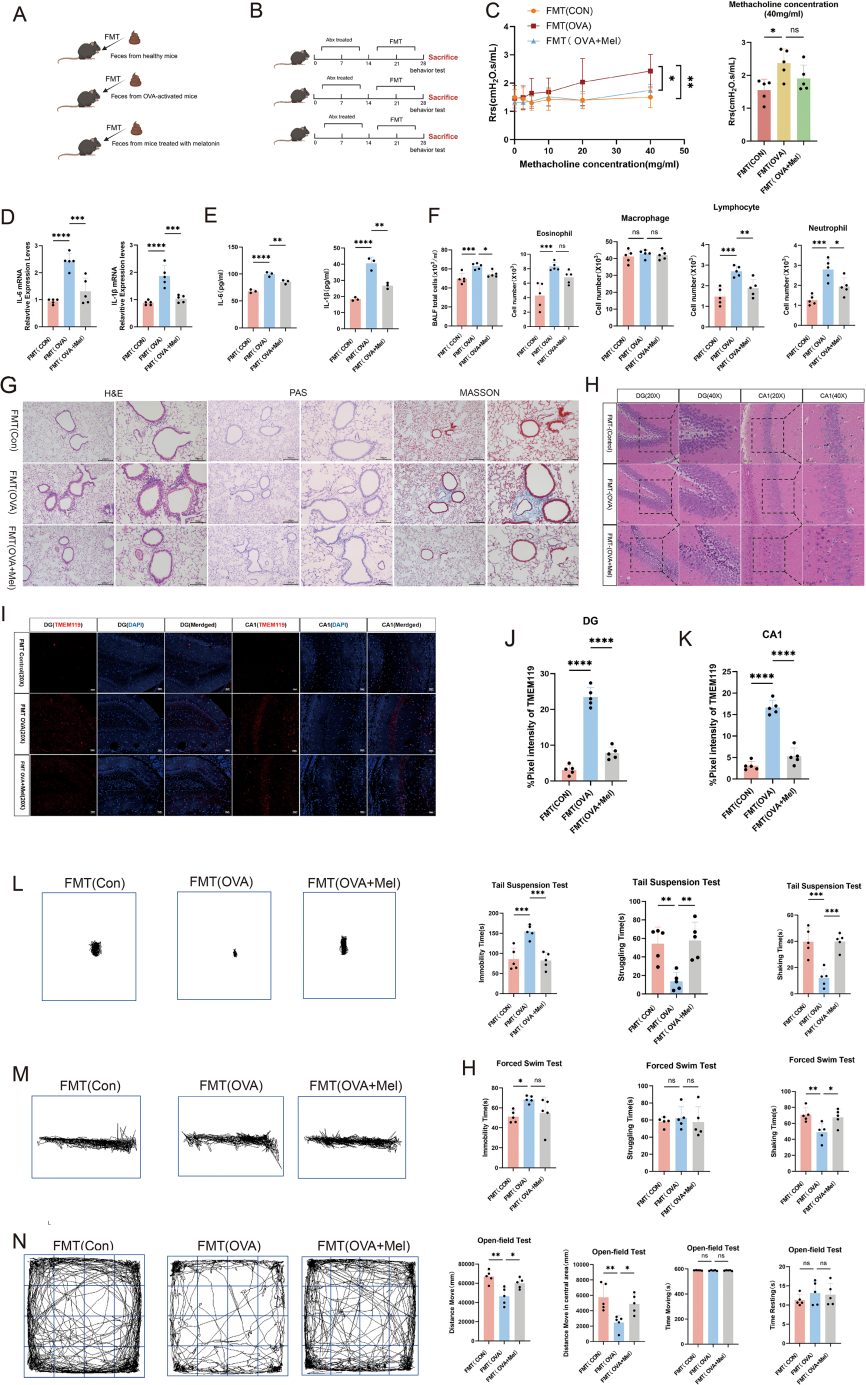
Fecal microbiota transplantation influences airway inflammation and anxiety-depressive behaviors in mice. **(A, B)** Schematic diagram of the fecal microbiota transplantation (FMT) procedure. **(C)** Airway responsiveness assessed by methacholine challenge test (40 mg/mL) (n=5). **(D)** mRNA levels of IL-6 and IL-1β in lung tissue measured by qPCR (n=5). **(E)** Protein levels of IL-1β and IL-6 in BALF measured by ELISA (n=3). **(F)** Total and differential inflammatory cell counts in BALF (n=5). **(G)** Representative H&E, PAS, and Masson’s trichrome staining of lung tissues (left scale bar: 200 μm; right scale bar: 100 μm). **(H)** Representative H&E staining of the hippocampal dentate gyrus (DG) and CA1 region (20X scale bar:100μm; 40X scale bar: 50 μm). **(I-K)** Representative immunofluorescence staining and quantitative analysis of TMEM119 in the DG and CA1 regions (scale bar: 50 μm). **(L, M)** Representative movement trajectories and quantification of immobility, struggling, and swinging time in TST and FST (n=5). **(N)** Representative movement trajectories and quantification of total distance, center distance, movement time, and rest time in OFT (n=5). Data are presented as mean ± SEM. ns, not significant (*p* > 0.05), *p < 0.05, **p < 0.01, ***p < 0.001, ****p < 0.0001.

To determine whether alterations in gut microbiota composition represent a causative factor in the observed phenotypes, we ablated the gut microbiota using an antibiotic cocktail. Following long-term antibiotic treatment, ABX-treated mice received FMT from OVA-induced asthmatic mice or melatonin-treated mice (**Figures 5A, B**).

Two weeks after FMT, mice receiving OVA-derived microbiota exhibited increased airway resistance compared to those receiving control or melatonin-treated microbiota (**Figure 5C**). Meanwhile, total and differential inflammatory cell counts in BALF were significantly elevated in these mice (with the exception of macrophages), along with markedly upregulated expression of the inflammatory cytokines IL-6 and IL-1β (**Figures 5D–F**). However, although the numbers of eosinophils and macrophages decreased following treatment, these changes did not reach statistical significance. Histological analysis further revealed enhanced inflammatory cell infiltration and increased pathological collagen deposition, but no observable hyperplasia, in the lung tissues of OVA microbiota recipients (**Figure 5G**).

Behaviorally, mice receiving OVA microbiota displayed depressive-like behaviors across multiple tests (**Figures 5L–N**). In contrast, mice transplanted with control or melatonin-treated microbiota showed no marked abnormalities in pulmonary inflammation or behavioral performance.

Further neurohistological examination indicated that OVA microbiota recipients exhibited thinning and disorganization of the cellular layer, along with vacuolar degeneration in the hippocampal CA1 region and dentate gyrus (**Figure 5G**). Additionally, increased expression of the microglial marker TMEM119 suggested enhanced microglial activation (**Figure 5H**).

Collectively, these data demonstrate that features of asthmatic airway inflammation and anxiety-depressive behaviors can be transferred to ABX-treated recipient mice through transplantation of gut microbiota from affected donors.

### Fecal microbiota transplantation from melatonin- or sodium butyrate-treated donors alleviates disease in asthmatic mice

**Figure 6:**
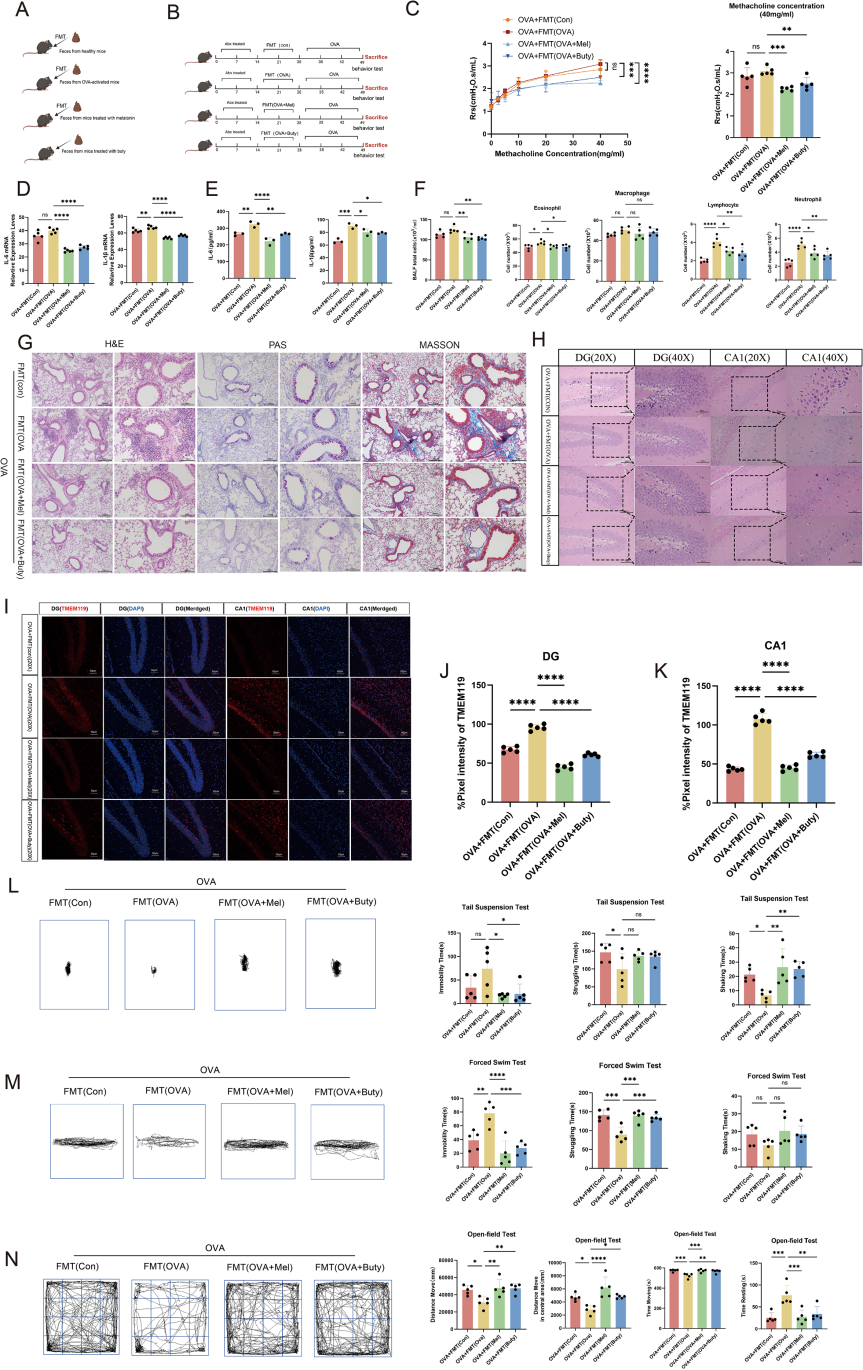
Fecal microbiota transplantation from melatonin- or sodium butyrate-treated donors alleviates disease in asthmatic mice. **(A, B)** Schematic diagram of the fecal microbiota transplantation (FMT) procedure. **(C)** Airway responsiveness assessed by methacholine challenge test (40 mg/mL) (n=5). **(D)** mRNA levels of IL-6 and IL-1β in lung tissue measured by qPCR (n=5). **(E)** Protein levels of IL-1β and IL-6 in BALF measured by ELISA (n=3). **(F)** Total and differential inflammatory cell counts in BALF (n=5). **(G)** Representative H&E, PAS, and Masson’s trichrome staining of lung tissues (left scale bar: 200 μm; right scale bar: 100 μm). **(H)** Representative H&E staining of the hippocampal DG and CA1 region (20X scale bar: 100μm; 40X scale bar: 50 μm). **(I-K)** Representative immunofluorescence staining and quantitative analysis of TMEM119 in the DG and CA1 regions (scale bar: 50 μm). **(L, M)** Representative movement trajectories and quantification of immobility, struggling, and swinging time in TST and FST (n=5). **(N)** Representative movement trajectories and quantification of total distance, center distance, movement time, and rest time in OFT (n=5). Data are presented as mean ± SEM. ns, not significant (*p* > 0.05), *p < 0.05, **p < 0.01, ***p < 0.001, ****p < 0.0001.

To investigate whether microbiota from melatonin- or sodium butyrate-treated donors alleviates OVA-induced asthma, we performed fecal microbiota transplantation (FMT) in an OVA-induced asthma model, transferring microbiota from melatonin-treated, sodium butyrate-treated, asthmatic, or healthy control donors (**Figures 6A, B**).

Mice colonized with microbiota from OVA-induced asthmatic donors exhibited a significantly exacerbated inflammatory phenotype compared to other groups, as evidenced by more severe airway inflammation (**Figure 6C**), elevated inflammatory cell counts in BALF, upregulated expression of IL-6 and IL-1β (**Figures 6D–F**), and pronounced pathological changes in lung tissue, including inflammatory infiltration and collagen deposition (**Figure 6G**). In contrast, mice receiving microbiota from melatonin- or sodium butyrate-treated donors showed significantly attenuated airway inflammation and reduced inflammatory cell infiltration relative to those receiving OVA-derived microbiota. Notably, transplantation of microbiota from healthy control donors resulted in inflammatory phenotypes similar to those of the standard OVA-induced asthma model, confirming that the FMT procedure itself did not alter baseline disease susceptibility and that the observed protective effects were specifically conferred by microbiota shaped by melatonin or sodium butyrate.

This pathological pattern extended to the central nervous system. Mice colonized with OVA-derived microbiota exhibited significant depressive-like behaviors (**Figures 6L–N**), along with hippocampal pathological changes and enhanced microglial activation (**Figures 6H–K**). By contrast, mice receiving microbiota from melatonin- or sodium butyrate-treated donors displayed partially attenuated neurobehavioral deficits and reduced microglial activation compared to those receiving OVA-derived microbiota, although their phenotypes did not fully return to normal levels. Mice that received microbiota from healthy control donors exhibited behavioral and neuropathological phenotypes comparable to those of the standard OVA-induced asthma model (**Figures 2**, **3**), confirming that the FMT procedure did not alter baseline susceptibility to neurobehavioral abnormalities and that the observed partial protective effects were specifically attributable to microbiota shaped by melatonin or sodium butyrate.

Taken together, these findings demonstrate that the gut microbiota is a critical mediator of both pulmonary and behavioral symptoms in asthma. Crucially, they show that the beneficial effects of melatonin and sodium butyrate can be partially transferred via FMT, alleviating airway inflammation and anxiety-depressive behaviors in recipient asthmatic mice, albeit without achieving complete restoration to healthy control levels.

### Melatonin alleviates airway inflammation and anxiety-depressive behaviors in asthmatic mice via the gut microbiota

**Figure 7:**
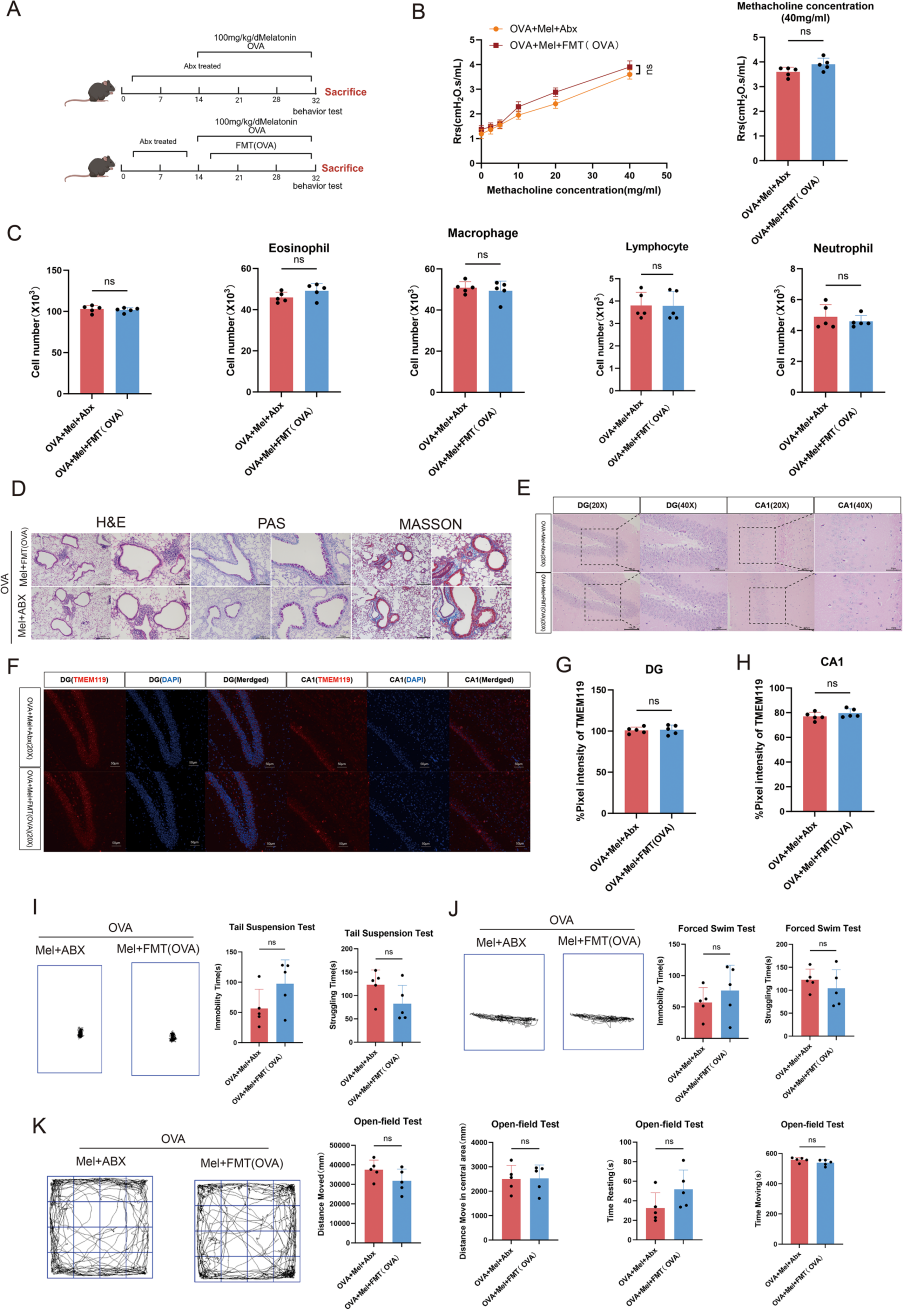
Gut microbiota depletion abrogates the therapeutic effects of melatonin on airway inflammation and anxiety-depressive behaviors in asthmatic mice. **(A)** Schematic diagram of the fecal microbiota transplantation (FMT) procedure. **(B)** Airway responsiveness assessed by methacholine challenge test (40 mg/mL) (n=5). **(C)** Total and differential inflammatory cell counts in BALF (n=5). **(D)** Representative H&E, PAS, and Masson’s trichrome staining of lung tissues (left scale bar: 200 μm; right scale bar: 100 μm). **(E)** Representative H&E staining of the hippocampal DG and CA1 region (20X scale bar: 100μm; 40X scale bar: 50μm). **(F-H)** Representative immunofluorescence staining and quantitative analysis of TMEM119 in the DG and CA1 regions (scale bar: 50 μm). **(I-J)** Representative movement trajectories and quantification of immobility, struggling, and swinging time in TST and FST (n=5). **(K)** Representative movement trajectories and quantification of total distance, center distance, movement time, and rest time in OFT (n=5). Data are presented as mean ± SEM. ns, not significant (*p* > 0.05).

To definitively establish the causal role of the gut microbiota in mediating melatonin’s therapeutic effects, we employed a combinatorial approach using antibiotic depletion and FMT (**Figure 7A**). Unlike the experiment in **Figure 6**, which tested whether the *beneficial* microbiota from melatonin-treated donors could transfer protection, this experiment was designed to test whether the gut microbiota is *necessary* for melatonin to exert its effects. Here, all mice first received antibiotics to ablate their native gut microbiota. Subsequently, they were subjected to the OVA-induced asthma protocol and received melatonin treatment. Crucially, during the challenge phase, these mice were also recolonized via FMT with microbiota from asthmatic donors, effectively replacing any melatonin-shaped microbes with a pathogenic community.

Under these conditions, where a healthy or melatonin-shaped microbiota was absent, melatonin failed to exert its protective effects. Results showed that after antibiotic-induced microbiota depletion and subsequent colonization with asthmatic microbiota, melatonin-treated mice exhibited no significant improvement in lung histopathology, including airway inflammation, mucus secretion, or collagen deposition (**Figure 7D**). Similarly, no marked differences were observed between groups in total and differential inflammatory cell counts in BALF (**Figure 7C**), or in airway resistance measured by lung function tests (**Figure 7B**).

At the behavioral level, both groups displayed comparable anxiety- and depressive-like behaviors (**Figures 7G–I**). Further histological examination of hippocampal tissues revealed similar degrees of neuronal damage in both groups, characterized by thinning and disorganization of the neuronal layer and vacuolar degeneration in the CA1 region and DG (**Figure 7E**). Additionally, increased expression of the microglial marker TMEM119 was observed in both groups (**Figure 7F**), indicating that neuroinflammatory status was not alleviated by melatonin intervention under microbiota-ablated conditions.

In summary, these findings underscore the essential role of the gut microbiota in mediating melatonin’s protective effects against asthma-related airway inflammation and anxiety-depressive behaviors, suggesting that melatonin’s therapeutic efficacy depends on the maintenance of a specific gut microbial ecological context.

### Melatonin/sodium butyrate/FMT alleviate anxiety-depressive behaviors in asthmatic mice via the MAPK/p65/NLRP3 pathway

**Figure 8:**
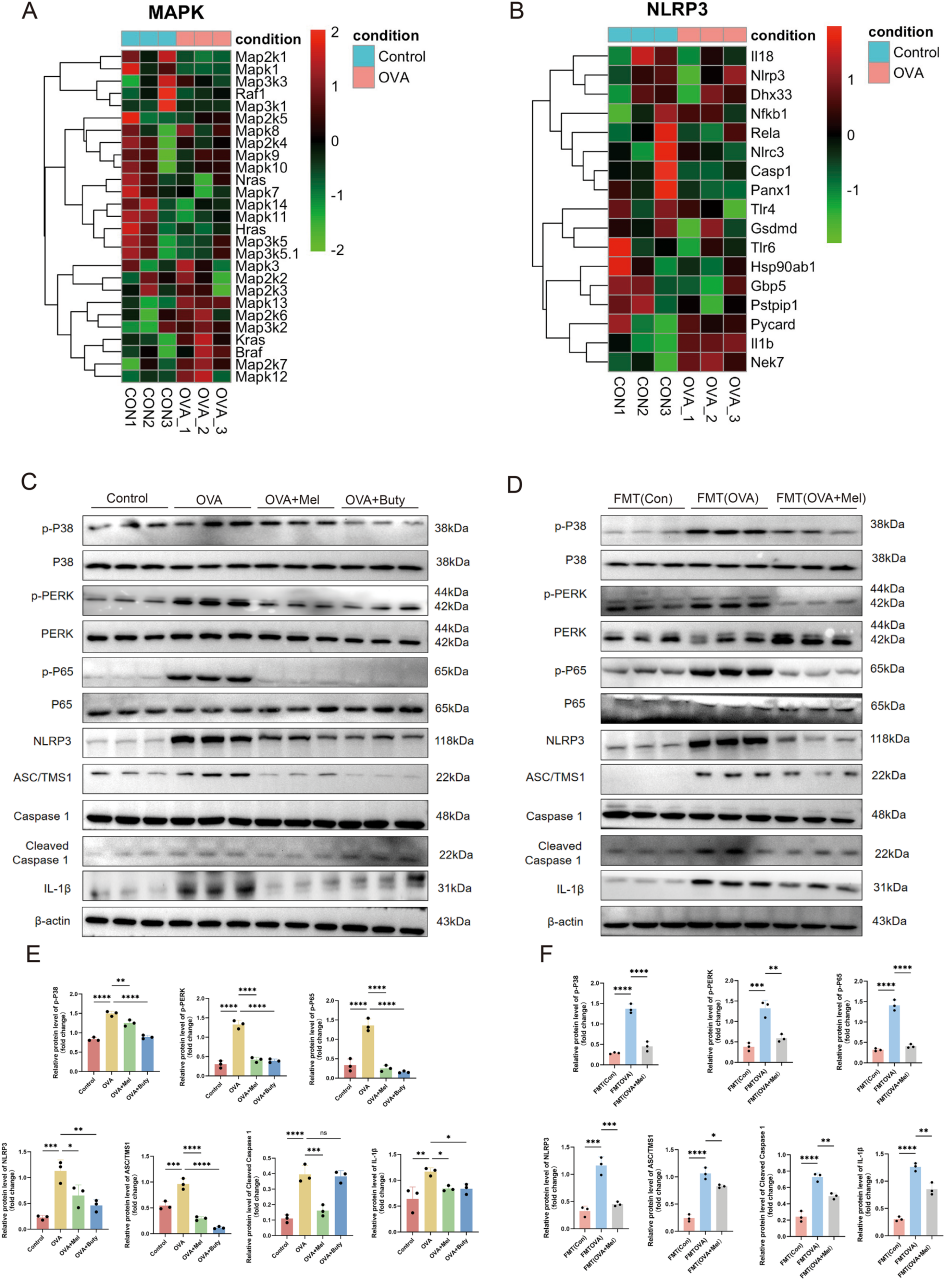
Melatonin/sodium butyrate/fecal microbiota transplantation alleviate anxiety and depression in asthmatic mice via the MAPK/P65/NLRP3 pathway. **(A, B)** Unbiased pathway analysis revealed significant differences in the activation of the MAPK signaling pathway and the NLRP3 inflammasome pathway. **(C, D)** Western blot analysis of key proteins in the MAPK/P65/NLRP3 signaling pathway in hippocampal tissues. **(E, F)** Quantitative analysis was performed on the protein expression levels of the MAPK/P65/NLRP3 signaling pathway in hippocampal tissue extracts from mice. Data are presented as mean ± SEM. ns, not significant (*p* > 0.05), *p < 0.05, **p < 0.01, ***p < 0.001, ****p < 0.0001.

To systematically elucidate the central mechanisms by which melatonin, sodium butyrate, and FMT improve anxiety-depressive behaviors in the asthma model, we first performed transcriptomic sequencing of microglia from brain tissue. Unbiased pathway analysis revealed significant differences in the activation of the MAPK signaling pathway and the NLRP3 inflammasome pathway (**Figures 8A, B**).

Based on these omics screening results, we conducted targeted validation of this MAPK/p65/NLRP3 signaling axis at the protein level. Western blot analysis clearly demonstrated that, compared with the control group, hippocampal tissues from asthmatic mice exhibited significantly increased protein levels of phosphorylated p38, phosphorylated pERK, and phosphorylated p65. All three interventions effectively suppressed the phosphorylation of these key signaling molecules (**Figures 8C–F**).

More importantly, downstream in this signaling pathway, we observed significantly upregulated protein expression of key components of the NLRP3 inflammasome (NLRP3 and ASC) and its functional output molecules—the activated forms of Caspase-1 and IL-1β—in the hippocampus of asthmatic mice. Melatonin, sodium butyrate, and FMT similarly significantly inhibited the expression of these molecules (**Figures 8C–F**).

In summary, melatonin, sodium butyrate, and FMT alleviate anxiety-depressive-like behaviors by suppressing excessive activation of the MAPK signaling pathway in the mouse brain, thereby inhibiting the assembly and activation of the downstream NLRP3 inflammasome and ultimately reducing neuroinflammation.

### Melatonin and sodium butyrate attenuate BV2 microglial activation via the MAPK/P65/NLRP3 pathway

**Figure 9:**
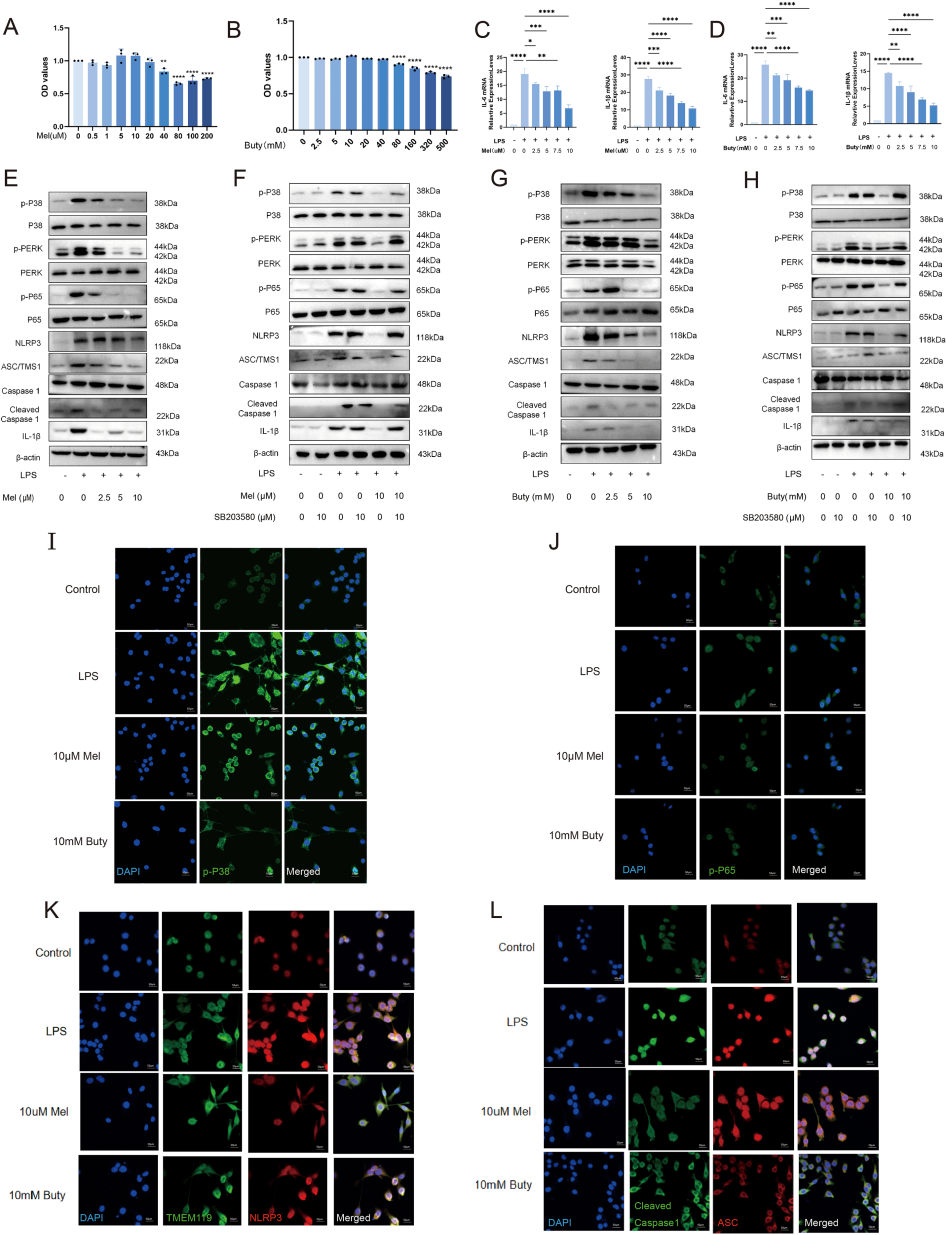
Melatonin and sodium butyrate alleviate BV2 microglial activation through the MAPK/P65/NLRP3 pathway. **(A, B)** Cytotoxicity tests of melatonin and sodium butyrate. **(C, D)** qPCR analysis of IL-6 and IL-1β mRNA levels in BV2 cells. **(E-H)** Protein blots of the MAPK/P65/NLRP3 pathway. **(I-L)** Immunofluorescence images of the MAPK/P65/NLRP3 pathway. (scale bar: 50 μm).Data are presented as mean ± SEM. *p < 0.05, **p < 0.01, ***p < 0.001, ****p < 0.0001.

To validate the regulatory effects of melatonin and sodium butyrate on the MAPK/P65/NLRP3 pathway at the cellular level, we established an LPS-induced inflammatory model in BV2 microglial cells. CCK-8 assays showed that neither melatonin (2.5–10 μM) nor sodium butyrate (2.5–10 mM) exhibited significant cytotoxicity toward BV2 cells (**Figures 9A, B**). qPCR results revealed that LPS stimulation markedly increased the mRNA levels of the inflammatory factors IL-6 and IL-1β, and these increases were concentration-dependently suppressed by pretreatment with either melatonin or sodium butyrate (**Figures 9C, D**).

At the protein level, Western blot analysis demonstrated that LPS significantly enhanced the phosphorylation of p-p38, p-ERK, and p-p65 in BV2 cells, whereas pretreatment with melatonin or sodium butyrate effectively inhibited their activation (**Figures 9E, G**). Concurrently, the expression of NLRP3 inflammasome components (NLRP3, ASC) and their downstream molecules (cleaved Caspase-1, mature IL-1β) was also significantly elevated following LPS stimulation, and these changes were markedly reversed by pretreatment with melatonin or sodium butyrate (**Figures 9E, G**).

To confirm the pivotal role of the MAPK pathway, we employed the p38 inhibitor SB203580. Results showed that SB203580 alone inhibited LPS-induced upregulation of p-p38, p-p65, and NLRP3 inflammasome activation, and no additive effect was observed when it was combined with melatonin or sodium butyrate (**Figures 9F, H**). Immunofluorescence staining further verified that LPS stimulation significantly increased the proportion of TMEM119-positive cells and induced morphological activation features in BV2 cells, whereas treatment with melatonin or sodium butyrate substantially suppressed microglial activation (**Figures 9I–L**).

These findings indicate that melatonin and sodium butyrate effectively attenuate LPS-induced BV2 microglial activation and neuroinflammatory responses by inhibiting the MAPK/P65/NLRP3 signaling pathway.

## Discussion

Asthma, a multifaceted chronic inflammatory condition of the airways, frequently coexists with neuropsychiatric disorders, including anxiety and depression, thus forming a systemic condition interconnected through the “lung-brain axis” (6, 25). Although the clinical association is well-established, the shared molecular mechanisms remain unclear. This study reveals that melatonin concurrently alleviates asthma-related airway inflammation and anxiety-depressive-like behaviors by modulating the “gut microbiota-short-chain fatty acid (SCFA)” axis and systemically inhibiting the MAPK/NLRP3 inflammatory pathway. This finding provides a novel perspective for understanding the integrated mechanisms linking asthma and its neuropsychiatric comorbidities and offers a theoretical basis for therapeutic strategies targeting the gut microbiota.

The gut microbiota and its metabolites, particularly SCFAs, serve as pivotal mediators in gut–organ communication, playing a crucial role in sustaining immune homeostasis and nervous system function (10, 26, 27). A substantial body of research has documented dysbiosis of the gut microbiota and a reduction in SCFA production in both human asthmatics and animal models (7, 28). The present study further clarifies that SCFA deficiency is not only a characteristic feature of asthmatic airway inflammation but also a pathological hub connecting it with comorbid emotional disorders. By exogenously supplementing with sodium butyrate, we successfully recapitulated the protective effects of melatonin; conversely, antibiotic-induced microbiota depletion completely abrogated melatonin’s therapeutic benefits. This causal chain of evidence establishes the “microbiota-SCFA axis” as a central mediator in the systemic effects of melatonin.

At the mechanistic level, we focused on the MAPK/NLRP3 inflammatory pathway. This pathway serves as a master regulator of cellular stress and inflammatory responses; its hyperactivation can drive NF-κB nuclear translocation and NLRP3 inflammasome assembly, contributing to various chronic inflammatory diseases (29). In this study, hippocampal tissues from asthmatic mice exhibited significantly elevated phosphorylation levels of p38 MAPK, ERK, and p65, along with upregulated expression of NLRP3, ASC, Caspase-1, and IL-1β. Melatonin, sodium butyrate, and FMT all markedly inhibited the activation of these molecules. Further *in vitro* experiments confirmed that melatonin and sodium butyrate dose-dependently suppressed the LPS-induced activation of the MAPK/P65/NLRP3 pathway in BV2 microglial cells. The p38 inhibitor SB203580 mimicked these effects, suggesting that this pathway is a key downstream target through which the melatonin-SCFA axis exerts its anti-inflammatory actions.

Notably, butyrate, as an HDAC inhibitor, can both alleviate pulmonary inflammation by promoting Treg differentiation and inhibiting the NF-κB pathway (30, 31) and directly modulate microglial function, promote BDNF expression, and inhibit HPA axis hyperactivation by crossing the blood-brain barrier (32). In this study, melatonin intervention significantly increased intestinal butyrate levels, which was accompanied by reduced hippocampal microglial activation, attenuated neuronal damage, and improved behavioral outcomes, further supporting butyrate’s role as a key mediator in the bidirectional “lung-brain” regulation.

The FMT experiments provided crucial evidence for causal inference in this study. Transplantation of fecal microbiota from melatonin-treated donor mice into asthmatic recipients significantly alleviated airway inflammation and anxiety-depressive-like behaviors, whereas transplantation of microbiota from asthmatic mice induced similar pathological phenotypes in recipient mice. Conversely, the protective effects of melatonin were completely abolished following antibiotic-induced microbiota depletion. These experiments collectively demonstrate that melatonin’s therapeutic effects are dependent on the presence and metabolic function of the gut microbiota, rather than resulting from direct action on the lungs or brain.

Based on the above evidence, we propose the following integrated mechanistic model: Under asthmatic conditions, gut microbiota dysbiosis leads to insufficient SCFA production, relieving the inhibition of the MAPK/NLRP3 pathway. This, on one hand, exacerbates pulmonary inflammation and, on the other hand, weakens anti-inflammatory and neurotrophic signals to the central nervous system, ultimately forming a lung-brain comorbidity. Melatonin restores the gut microbial structure and elevates SCFA levels, thereby systemically inhibiting the MAPK/NLRP3 inflammatory axis and achieving dual protection for both the lungs and the brain.

This study has several limitations. First, transcriptomic sequencing was performed on microglia isolated from the whole brain, which may have diluted region-specific signals from the hippocampus; future studies should employ region-specific analytical methods for further validation. Second, although the BV2 cell line is widely used in neuroinflammation research, the LPS stimulation model cannot fully recapitulate the neuroinflammatory microenvironment associated with type 2-dominant asthma. Third, the specific role of SCFA receptors in mediating these effects remains to be clarified using gene knockout animal models. Fourth, it is difficult to definitively distinguish whether the observed behavioral improvements result from direct central effects or are secondary to the alleviation of respiratory symptoms. Future studies should employ behavioral paradigms less dependent on locomotor activity or monitor respiratory function concurrently during behavioral testing to further disentangle causal pathways. Fifth, the precise mechanisms by which melatonin regulates the gut microbiota require further investigation. Finally, the translational potential of these preclinical findings needs to be validated in human asthma populations.

## Conclusion

Melatonin may alleviate asthma-related airway inflammation and comorbid anxiety-depressive behaviors in mice, potentially through modulation of the gut microbiota-SCFA axis and suppression of hippocampal MAPK/P65/NLRP3 signaling. These findings provide a preliminary mechanistic framework that may inform future therapeutic strategies for asthma and its associated neuropsychiatric comorbidities, although further validation is required.

## Data Availability

The datasets presented in this study can be found in online repositories. The names of the repository/repositories and accession number(s) can be found in the article/**Supplementary Material**.

## References

[1] Zissler UM Esser-von Bieren J Jakwerth CA Chaker AM Schmidt-Weber CB . Current and future biomarkers in allergic asthma. Allergy. (2016) 71:475–94. doi: 10.1111/all.12828. 26706728

[2] Bradding P Porsbjerg C Côté A Dahlén SE Hallstrand TS Brightling CE . Airway hyperresponsiveness in asthma: The role of the epithelium. J Allergy Clin Immunol. (2024) 153:1181–93. doi: 10.1016/j.jaci.2024.02.011. 38395082

[3] Miller RL Grayson MH Strothman K . Advances in asthma: New understandings of asthma's natural history, risk factors, underlying mechanisms, and clinical management. J Allergy Clin Immunol. (2021) 148:1430–41. doi: 10.1016/j.jaci.2021.10.001. 34655640

[4] Ye G Baldwin DS Hou R . Anxiety in asthma: a systematic review and meta-analysis. Psychol Med. (2021) 51:11–20. doi: 10.1017/S0033291720005097. 33431086

[5] Hurtado-Ruzza R Iglesias Ó Dacal-Quintas R Becerro-de-Bengoa-Vallejo R Calvo-Lobo C San-Antolín M . Asthma, much more than a respiratory disease: influence of depression and anxiety. Rev Assoc Med Bras (1992). (2021) 67:571–6. doi: 10.1590/1806-9282.20201066. 34495063

[6] Simões Cunha M Amaral R Pereira AM Almeida R Alves-Correia M Loureiro CC . Symptoms of anxiety and depression in patients with persistent asthma: a cross-sectional analysis of the INSPIRERS studies. BMJ Open. (2023) 13:e068725. doi: 10.1136/bmjopen-2022-068725. 37147092 PMC10163458

[7] Yu B Pei C Peng W Zheng Y Fu Y Wang X . Microbiota-derived butyrate alleviates asthma via inhibiting Tfh13-mediated IgE production. Signal Transduct Target Ther. (2025) 10:181. doi: 10.1038/s41392-025-02263-2. 40473603 PMC12141656

[8] Li Z Lai J Zhang P Ding J Jiang J Liu C . Multi-omics analyses of serum metabolome, gut microbiome and brain function reveal dysregulated microbiota-gut-brain axis in bipolar depression. Mol Psychiatry. (2022) 27:4123–35. doi: 10.1038/s41380-022-01569-9. 35444255

[9] Adak A Khan MR . An insight into gut microbiota and its functionalities. Cell Mol Life Sci. (2019) 76:473–93. doi: 10.1007/s00018-018-2943-4. 30317530 PMC11105460

[10] Tan JK Macia L Mackay CR . Dietary fiber and SCFAs in the regulation of mucosal immunity. J Allergy Clin Immunol. (2023) 151:361–70. doi: 10.1016/j.jaci.2022.11.007. 36543697

[11] Tagé BSS Gonzatti MB Vieira RP Keller AC Bortoluci KR Aimbire F . Three main SCFAs mitigate lung inflammation and tissue remodeling Nlrp3-dependent in murine HDM-induced neutrophilic asthma. Inflammation. (2024) 47:1386–402. doi: 10.1007/s10753-024-01983-x. 38329636

[12] van de Pol MA Lutter R Smids BS Weersink EJ van der Zee JS . Synbiotics reduce allergen-induced T-helper 2 response and improve peak expiratory flow in allergic asthmatics. Allergy. (2011) 66:39–47. doi: 10.1111/j.1398-9995.2010.02454.x. 20716319

[13] Parilli-Moser I Domínguez-López I Trius-Soler M Castellví M Bosch B Castro-Barquero S . Consumption of peanut products improves memory and stress response in healthy adults from the ARISTOTLE study: A 6-month randomized controlled trial. Clin Nutr. (2021) 40:5556–67. doi: 10.1016/j.clnu.2021.09.020. 34656952

[14] Madsen MT Zahid JA Hansen CH Grummedal O Hansen JR Isbrand A . The effect of melatonin on depressive symptoms and anxiety in patients after acute coronary syndrome: The MEDACIS randomized clinical trial. J Psychiatr Res. (2019) 119:84–94. doi: 10.1016/j.jpsychires.2019.09.014. 31586772

[15] Hansen MV Andersen LT Madsen MT Hageman I Rasmussen LS Bokmand S . Effect of melatonin on depressive symptoms and anxiety in patients undergoing breast cancer surgery: a randomized, double-blind, placebo-controlled trial. Breast Cancer Res Treat. (2014) 145:683–95. doi: 10.1007/s10549-014-2962-2. 24756186

[16] Habtemariam S Daglia M Sureda A Selamoglu Z Gulhan MF Nabavi SM . Melatonin and respiratory diseases: A review. Curr Top Med Chem. (2017) 17:467–88. doi: 10.2174/1568026616666160824120338, PMID: 27558675

[17] Shin IS Park JW Shin NR Jeon CM Kwon OK Kim JS . Melatonin reduces airway inflammation in ovalbumin-induced asthma. Immunobiology. (2014) 219:901–8. doi: 10.1016/j.imbio.2014.08.004. 25161126

[18] Jia Y Zhang T He M Yang B Wang Z Liu Y . Melatonin protects against colistin-induced intestinal inflammation and microbiota dysbiosis. J Pineal Res. (2024) 76:e12989. doi: 10.1111/jpi.12989. 38978438

[19] Mo X Shen L Wang X Ni W Li L Xia L . Melatonin mitigates sarcopenic obesity via microbiota and short-chain fatty acids: Evidence from epidemiologic and *in vivo* studies. J Cachexia Sarcopenia Muscle. (2025) 16:e13869. doi: 10.1002/jcsm.13869. 40511533 PMC12163512

[20] Huang D Sun C Chen M Bai S Zhao X Wang W . Bergenin ameliorates airway inflammation and remodeling in asthma by activating SIRT1 in macrophages to regulate the NF-κB pathway. Front Pharmacol. (2022) 13:994878. doi: 10.3389/fphar.2022.994878. 36313381 PMC9606584

[21] Xu Q Sun L Chen Q Jiao C Wang Y Li H . Gut microbiota dysbiosis contributes to depression-like behaviors via hippocampal NLRP3-mediated neuroinflammation in a postpartum depression mouse model. Brain Behav Immun. (2024) 119:220–35. doi: 10.1016/j.bbi.2024.04.002. 38599497

[22] He N Shen G Jin X Li H Wang J Xu L . Resveratrol suppresses microglial activation and promotes functional recovery of traumatic spinal cord via improving intestinal microbiota. Pharmacol Res. (2022) 183:106377. doi: 10.1016/j.phrs.2022.106377. 35926806

[23] Zhao Z Ning J Bao XQ Shang M Ma J Li G . Fecal microbiota transplantation protects rotenone-induced Parkinson's disease mice via suppressing inflammation mediated by the lipopolysaccharide-TLR4 signaling pathway through the microbiota-gut-brain axis. Microbiome. (2021) 9:226. doi: 10.1186/s40168-021-01107-9. 34784980 PMC8597301

[24] Li F Lai J Ma F Cai Y Li S Feng Z . Maternal melatonin supplementation shapes gut microbiota and protects against inflammation in early life. Int Immunopharmacol. (2023) 120:110359. doi: 10.1016/j.intimp.2023.110359. 37257272

[25] Licari A Castagnoli R Ciprandi R Brambilla I Guasti E Marseglia GL . Anxiety and depression in adolescents with asthma: a study in clinical practice. Acta BioMed. (2022) 93:e2022021. doi: 10.23750/ABM.V93I1.10731, PMID: 35315428 PMC8972864

[26] Mann ER Lam YK Uhlig HH . Short-chain fatty acids: linking diet, the microbiome and immunity. Nat Rev Immunol. (2024) 24:577–95. doi: 10.1038/s41577-024-01014-8. 38565643

[27] Doifode T Giridharan VV Generoso JS Bhatti G Collodel A Schulz PE . The impact of the microbiota-gut-brain axis on Alzheimer's disease pathophysiology. Pharmacol Res. (2021) 164:105314. doi: 10.1016/j.phrs.2020.105314. 33246175

[28] Yip W Hughes MR Li Y Cait A Hirst M Mohn WW . Butyrate shapes immune cell fate and function in allergic asthma. Front Immunol. (2021) 12:628453. doi: 10.3389/fimmu.2021.628453. 33659009 PMC7917140

[29] Gan P Gao Z Zhao X Qi G . Surfactin inducing mitochondria-dependent ROS to activate MAPKs, NF-κB and inflammasomes in macrophages for adjuvant activity. Sci Rep. (2016) 6:39303. doi: 10.1038/srep39303. 27966632 PMC5155226

[30] Wen B Huang Y Deng G Yan Q Jia L . Gut microbiota analysis and LC-MS-based metabolomics to investigate AMPK/NF-κB regulated by Clostridium butyricum in the treatment of acute pancreatitis. J Transl Med. (2024) 22:1072. doi: 10.1186/s12967-024-05764-w. 39604956 PMC11600808

[31] Carey ST Gammon JM Jewell CM . Biomaterial-enabled induction of pancreatic-specific regulatory T cells through distinct signal transduction pathways. Drug Delivery Transl Res. (2021) 11:2468–81. doi: 10.1007/s13346-021-01075-5. 34611846 PMC8581478

[32] Wei H Yu C Zhang C Ren Y Guo L Wang T . Butyrate ameliorates chronic alcoholic central nervous damage by suppressing microglia-mediated neuroinflammation and modulating the microbiome-gut-brain axis. BioMed Pharmacother. (2023) 160:114308. doi: 10.1016/j.biopha.2023.114308. 36709599

