# Supplementary material for: Melatonin alleviates airway inflammation and anxiety-depression in asthma via gut microbiota–SCFA axis-mediated inhibition of microglial activation
Source: Front Immunol. 2026 Mar 11;17:1763305. doi: 10.3389/fimmu.2026.1763305 (PMC13013491; doi:10.3389/fimmu.2026.1763305)
Supplement: Supplementary file 1 [file Table1.docx]

**Table S1. Daily monitoring of water intake**

| **Experimental Day** | **Control** | **OVA** | **OVA+Mel** | **OVA+Buty** |
| --- | --- | --- | --- | --- |
| Water intake/ml/Cage/Day | 60 | 60 | 60 | 60 |
| Day 1 | 51 | 49 | 47 | 52 |
| Day 2 | 49 | 48 | 55 | 53 |
| Day 3 | 50 | 50 | 53 | 49 |
| Day 4 | 53 | 49 | 48 | 54 |
| Day 5 | 48 | 51 | 49 | 47 |
| Day 6 | 50 | 48 | 53 | 49 |
| Day 7 | 47 | 51 | 53 | 50 |
| Day 8 | 49 | 50 | 52 | 52 |
| Day 9 | 50 | 48 | 54 | 51 |
| Day 10 | 47 | 49 | 52 | 49 |
| Day 11 | 51 | 52 | 51 | 48 |
| Day 12 | 49 | 50 | 50 | 50 |
| Day 13 | 54 | 53 | 49 | 51 |
| Day 14 | 50 | 49 | 48 | 52 |
| Day 15 | 49 | 52 | 49 | 50 |
| Day 16 | 54 | 51 | 50 | 49 |
| Day 17 | 51 | 54 | 51 | 48 |
| Day 18 | 49 | 50 | 52 | 49 |
| Day 19 | 51 | 48 | 54 | 50 |
| Day 20 | 54 | 53 | 53 | 54 |
| Day 21 | 52 | 51 | 51 | 52 |

**Table S2. Daily weight**

| **Experimental Day** | **Control** | **OVA** | **OVA+Mel** | **OVA+Buty** |
| --- | --- | --- | --- | --- |
| weight (g, mean ± SD) |  |  |  |  |
| Day 1 | 21.3 ± 0.4 | 21.3 ± 0.4 | 21.3 ± 0.4 | 21.3 ± 0.4 |
| Day 2 | 21.4 ± 0.4 | 21.3 ± 0.4 | 21.4 ± 0.4 | 21.3 ± 0.4 |
| Day 3 | 21.6 ± 0.4 | 21.5 ± 0.4 | 21.5 ± 0.4 | 21.5 ± 0.4 |
| Day 4 | 21.6± 0.4 | 21.4± 0.4 | 21.6± 0.4 | 21.4± 0.4 |
| Day 5 | 21.9 ± 0.4 | 21.6 ± 0.4 | 21.5 ± 0.4 | 21.6 ± 0.4 |
| Day 6 | 21.8 ± 0.4 | 21.5 ± 0.4 | 21.7 ± 0.4 | 21.5 ± 0.4 |
| Day 7 | 22.3 ± 0.4 | 21.8 ± 0.4 | 21.9 ± 0.4 | 21.7± 0.4 |
| Day 8 | 21.9 ± 0.4 | 21.9 ± 0.4 | 21.9 ± 0.4 | 21.8 ± 0.4 |
| Day 9 | 22.3 ± 0.4 | 22.0 ± 0.4 | 22.0± 0.4 | 22.0± 0.4 |
| Day 10 | 22.4 ± 0.4 | 22.1 ± 0.4 | 22.2 ± 0.4 | 22.1 ± 0.4 |
| Day 11 | 22.5± 0.4 | 22.0± 0.4 | 22.3± 0.4 | 22.1± 0.4 |
| Day 12 | 22.4 ± 0.4 | 22.3± 0.4 | 22.4 ± 0.4 | 22.2 ± 0.4 |
| Day 13 | 22.7 ± 0.4 | 22.4 ± 0.4 | 22.5 ± 0.4 | 22.1 ± 0.4 |
| Day 14 | 22.7 ± 0.4 | 22.5 ± 0.4 | 22.3 ± 0.4 | 22.2 ± 0.4 |
| Day 15 | 22.8 ± 0.4 | 22.3± 0.4 | 22.5 ± 0.4 | 22.3 ± 0.4 |
| Day 16 | 22.8 ± 0.4 | 22.5 ± 0.4 | 22.6 ± 0.4 | 22.2 ± 0.4 |
| Day 17 | 23.2 ± 0.4 | 22.8 ± 0.4 | 22.9 ± 0.4 | 22.5± 0.4 |
| Day 18 | 23.3 ± 0.4 | 22.9 ± 0.4 | 23.0 ± 0.4 | 22.6 ± 0.4 |
| Day 19 | 23.3 ± 0.4 | 23.0 ± 0.4 | 23.1 ± 0.4 | 22.9 ± 0.4 |
| Day 20 | 23.4 ± 0.4 | 22.9 ± 0.4 | 23.2 ± 0.4 | 23.1± 0.4 |
| Day 21 | 23.3 ± 0.4 | 23.0 ± 0.4 | 23.1 ± 0.4 | 23.2 ± 0.4 |
