# Supplementary material for: Melatonin alleviates airway inflammation and anxiety-depression in asthma via gut microbiota–SCFA axis-mediated inhibition of microglial activation
Source: Front Immunol. 2026 Mar 11;17:1763305. doi: 10.3389/fimmu.2026.1763305 (PMC13013491; doi:10.3389/fimmu.2026.1763305)
Supplement: Supplementary file 2 [file Table2.docx]

****Supplementary Fig. S1. Nissl staining of the mPFC and hippocampal DG.**** Representative images showing neuronal morphology in the medial prefrontal cortex (mPFC) and dentate gyrus (DG) from each group. Scale bar: 50 μm. n = 5 per group.

****Supplementary Fig. S2.Gut microbiota compositional analysis.****(A) NMDS plot based on Bray-Curtis distances showing microbial community structure (n=5). (B) ANOSIM test results revealing significant separation between groups. (C) LEfSe cladogram and LDA score plot identifying differentially abundant taxa enriched in each group.
