# Supplementary figures and images for: Melatonin alleviates airway inflammation and anxiety-depression in asthma via gut microbiota–SCFA axis-mediated inhibition of microglial activation
Source: Front Immunol. 2026 Mar 11;17:1763305. doi: 10.3389/fimmu.2026.1763305 (PMC13013491; doi:10.3389/fimmu.2026.1763305)

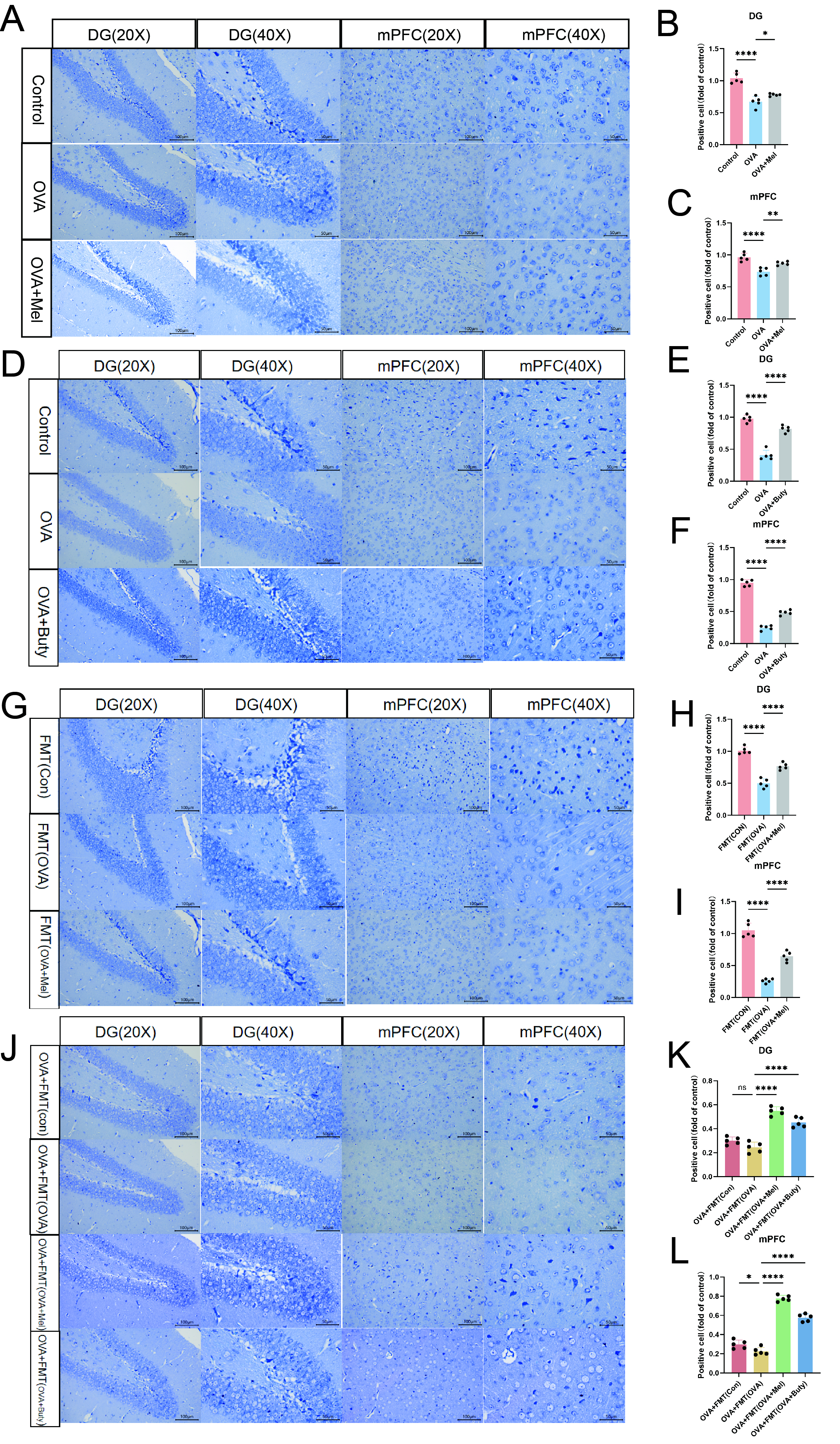

Supplement: Supplementary file 3 [file Image1.jpeg]

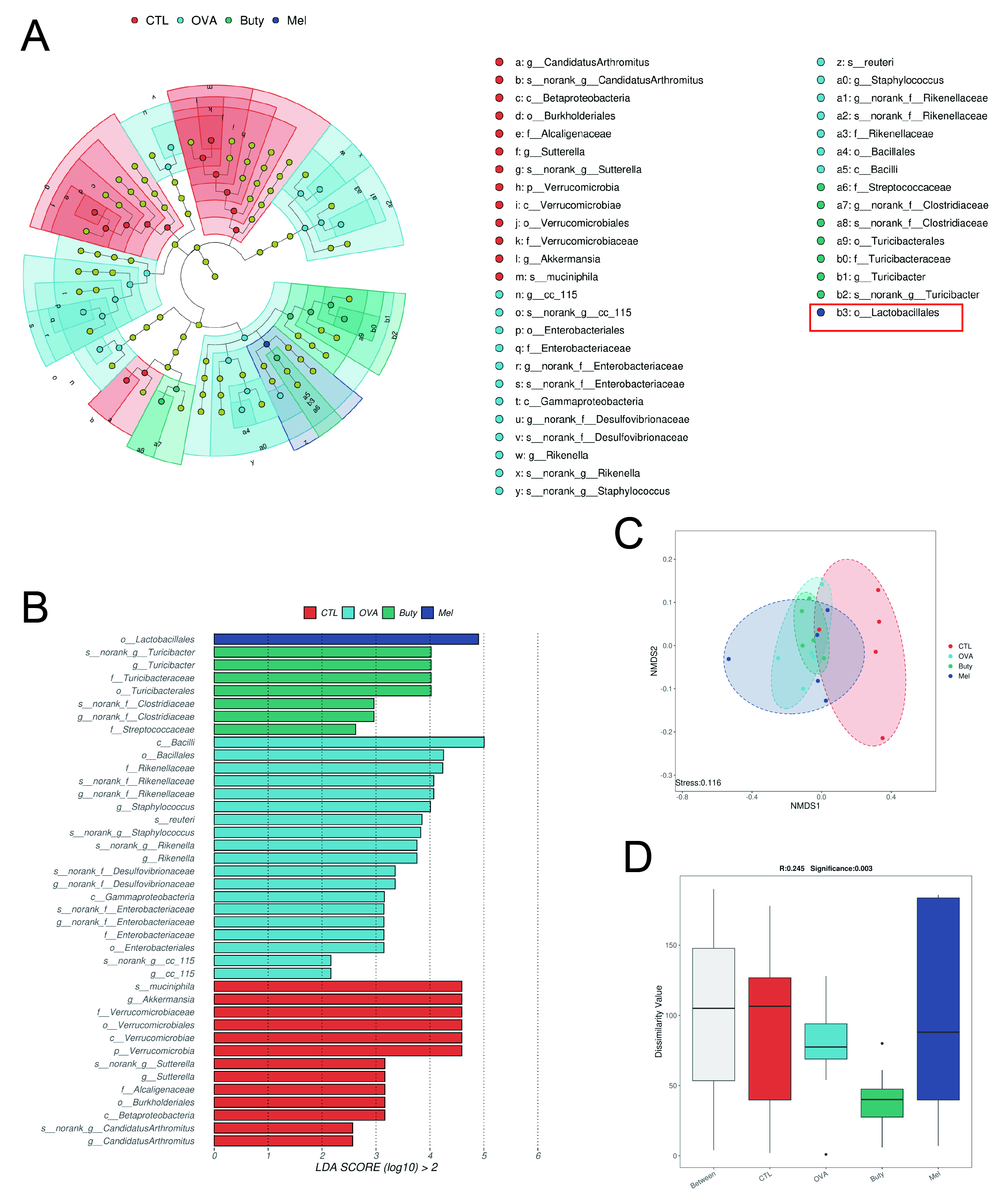

Supplement: Supplementary file 4 [file Image2.tif]
